# Landscape of flavonoid metabolism in human gut microbiome and its association with health and disease

**DOI:** 10.1080/29933935.2025.2520788

**Published:** 2025-06-25

**Authors:** Deepika Pateriya, Vishnu Prasoodanan P. K., Joy Scaria, Vineet K. Sharma

**Affiliations:** a MetaBioSys Group, Department of Biological Sciences, Indian Institute of Science Education and Research Bhopal, Bhopal, India; b Department of Veterinary Pathobiology, College of Veterinary Medicine, Oklahoma State University, Stillwater, OK, USA

**Keywords:** Disease, flavonoid, gut microbiome, health, metabolism

## Abstract

The positive effects of dietary flavonoids on health depend on their bioavailability in the human gut, where the flavonoid-modifying enzymes (FMEs) in gut bacteria play a crucial role in flavonoid metabolism. Thus, to comprehensively examine the role of FMEs in this process, we first constructed a database of potential FMEs containing 6,865 proteins. We identified homologs of these FMEs in gut bacterial genomes and reported species that can potentially modify flavonoids but were not previously known in this context. We examined the differential abundance of FMEs in the gut microbiomes of healthy and diseased individuals from Western and non-Western populations with distinct dietary habits. The differential enrichment of key FMEs between Western and non-Western populations and between disease and healthy samples highlights differences in gut flavonoid metabolism based on diet, population, and health status. This study reveals a comprehensive landscape of flavonoid metabolism in the human gut microbiome.

## GRAPHICAL ABSTRACT

**Figure:**
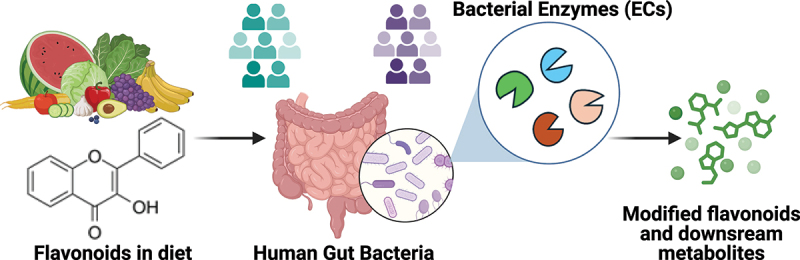

## Introduction

The human gut microbiome plays a crucial role in the metabolism of dietary components by contributing a wide range of enzymatic proteins not encoded by the host genome. Flavonoids are polyphenolic plant secondary metabolites, commonly present in multiple dietary plant sources, such as fruits, vegetables, herbs, seeds, cereals, and beverages.^1^ The basic structure of flavonoids comprises an aromatic A ring attached to a heterocyclic C ring, which binds to the aromatic C ring.^1^ Based on their chemical structure, flavonoids are categorized into multiple classes, including flavones, flavonols, flavanones, flavanonols, isoflavones, and anthocyanins.^1^ After consumption, a portion of flavonoids is absorbed in the small intestine following O-deglycosylation by epithelial enzymes. Subsequently, in the intestinal epithelium cells and liver, phase I and phase II transformation of flavonoid aglycons takes place.^2–4^

A large part of dietary flavonoids (or their phase I/II metabolites) reaches the large intestine, where they are subject to transformation by gut bacteria. There are various bacterial enzymes reported to carry out flavonoid modification in the large intestine, including decarboxylation, ring cleavage, reduction, isomerization, and demethylation.^5^ Most dietary flavonoids are present in the form of their glycosides.^6^ Gut bacterial alpha-L-rhamnosidase carries out the hydrolysis of terminal alpha-L-rhamnose from plant glycosides, including flavonoid glycosides, converting them into glucosides.^7–11^ Bacterial beta-glucosidases deglycosylate flavonoids, yielding absorbable aglycons, which could be absorbed in the large intestine or further metabolized into a variety of ring fission products.^12–15^

A well-studied pathway in bacterial-mediated flavonoid metabolism in the gut is the equol biosynthesis pathway, which involves key enzymes that convert isoflavone aglycons to equol.^16^ Another well-studied flavonoid metabolism pathway involves the degradation of flavones/flavonols where *Flavonifractor plautii* and *Eubacterium ramulus* are active species in flavonoid conversion.^17,18^ Reported enzymes in these species include Flavone/flavonol reductase (Flr), chalcone isomerase (Chi), Flavanone/flavanonol-cleaving reductase (Fcr), and phloretin hydrolase (Phy).^19–22^

Flavonoids and their metabolites have been reported to display a multitude of beneficial effects on human health due to their anti-inflammatory, antioxidant, and anti-cancerogenic properties.^23,24^ The protective effects of flavonoids have been reported in various diseases, including colorectal cancer (CRC) and inflammatory bowel disease (IBD).^25–27^ Flavonoids exert anticancerous effects in different ways, such as inhibition of cell proliferation, affecting inflammatory signaling pathways, inducing apoptosis, and inhibiting cancer cell growth.^28–40^

While these studies have highlighted the beneficial effects of flavonoids and their metabolites,^25,33^ our understanding of how the gut microbiome modifies flavonoids in individuals still remains limited since a large fraction of the flavonoid-modifying enzymes have been identified and characterized only in recent years.^5^ Since flavonoid metabolism is largely carried out by the gut bacteria, the abundance of flavonoid-modifying microbial enzymes and resultant beneficial flavonoid metabolites may vary with the composition of a healthy individual’s gut microbiome^41^ or due to the dysbiosis of gut microbial communities, such as in IBD and CRC.^42,43^ Furthermore, it is essential to identify and analyze the diversity of the yet unknown flavonoid-modifying enzymes and gut microbial species harboring them in different demographic groups having dietary variations.^5,41^

Thus, we first constructed a comprehensive database of flavonoid-modifying enzymes (FMEs) containing 6,865 potential flavonoid-modifying proteins from the bacterial species. We examined differentially abundant genes encoding these FMEs in the gut microbiome of healthy populations with diverse dietary habits, including populations with Western diets (US, Netherlands, Austria, China, Italy) and those with non-Western diets (India, Peru, Tanzania, Madagascar). We further investigated the differentially abundant genes encoding these FMEs in the gut microbiome of IBD and CRC patients. Our analysis unveils enzymes within the gut microbiome that have not been previously reported in the context of flavonoid metabolism but have the potential to modify flavonoids. Furthermore, our findings offer valuable insights into the differences in flavonoid-modifying capacity among healthy and diseased individuals with diverse dietary habits.

## Results

### Identification of potential FME and their homologs in human gut bacteria

We employed a comprehensive approach to identify potential FMEs from the literature. The query using flavonoid-related keywords in biochemical databases, including the Kyoto Encyclopedia of Genes and Genomes (KEGG) and Braunschweig Enzyme Database (BRENDA), identified 232 Enzyme Commission (EC) numbers with 3,92,775 protein sequences that can utilize various flavonoids as substrates. The 60 biochemically characterized FMEs involved in gut bacterial flavonoid metabolism from published literature were also included (Supplementary Table S1). After removing eukaryotic sequences and redundant sequences 30,601 prokaryotic sequences corresponding to 95 ECs were obtained, while the EC numbers for 41 biochemically characterized FMEs were not available.

**Figure 1.**
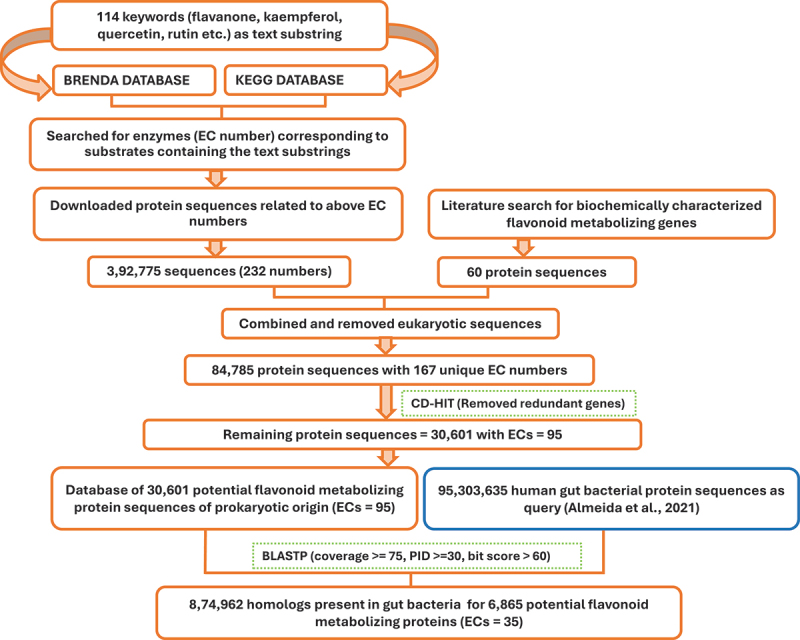
Workflow for identifying potential flavonoid-modifying enzymes (FMEs) in human gut bacterial genomes.

**Figure 2.**
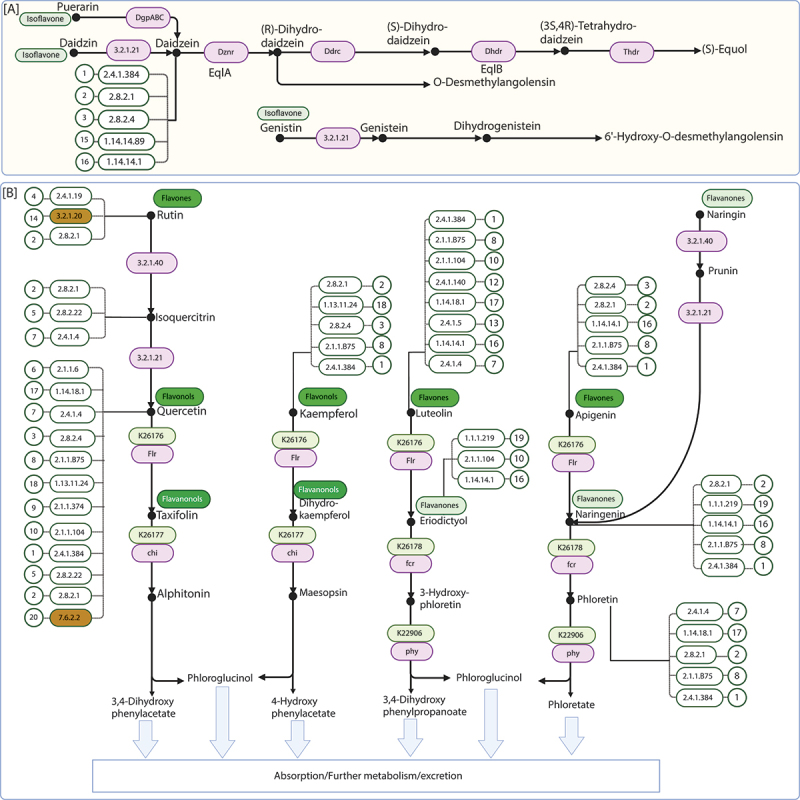
The figure illustrates two major pathways of flavonoid metabolism sourced from the KEGG database (map00946) along with ECs that have the potential to act upon substrates mentioned in these pathways. (A) Depicts the equol biosynthesis pathway. (B) Shows the flavonol/flavone degradation pathway. Previously reported enzymes in map00946 are highlighted in pink. Previously unreported ECs potentially involved in flavonoid metabolism, identified in this study are depicted without color and connected by dotted lines to their potential substrates. Two of these ECs (7.6.2.2 and 3.2.1.20) are separately highlighted in yellow, since these are not reported to modify but may interact with flavonoid substrates. Numbers associated with each dotted line correspond to detailed information about these ECs in Table 1.

To identify gut bacterial enzymes with flavonoid-modifying potential, homologs of potential FMEs in the Unified Human Gastrointestinal Protein (UHGP) catalog (UHGP-100, 2021–12–07) were identified. Protein sequences from the UHGP catalog were aligned against the custom database of 30,601 prokaryotic FMEs using Diamond software v2.0.15.153. A total of 6,865 bacterial protein sequences with 8,74,962 homologs in the UHGP database were identified, which represents potential FMEs in human gut bacteria (Figure 1). These 6,865 sequences correspond to 35 ECs and include 41 biochemically characterized FMEs lacking EC numbers. Of the 35 ECs, 26 ECs were not previously reported in bacteria-mediated flavonoid metabolism in the gut. The potential substrates for these enzymes are highlighted in Table 1, Supplementary Table S2, and Figure 2. A website providing key details of these FMEs was developed and is freely accessible at https://metabiosys.iiserb.ac.in/pfme.

**Table 1.** Summary of potential flavonoid-modifying ECs.

EC	Enzyme Name	Literature Genes	UHGG Species Count	Modification Type	Flavonoid Substrates	Previously reported in flavonoid modification in the Human Gut	Potential link with substrates in Figure 2
2.4.1.140	alternansucrase	1	1	Modification	luteolin, myricetin, quercetin	No	12
2.8.2.4	estrone sulfotransferase	1	1	Modification	apigenin, daidzein, kaempferol, quercetin	No	3
1.14.12.12_1.14.12.23_1.14.12.24	naphthalene 1,2-dioxygenase	2	2	Cleavage	(2S) flavanone, (3S)-isoflavanone	No	NA
2.1.1.6	catechol O-methyltransferase	3	3	Modification	catechin, procyanidin dimer, quercetin	No	6
2.1.1.374	2-heptyl-1-hydroxyquinolin-4(1 h)-one methyltransferase	10	5	Modification	quercetin	No	9
1.14.18.1	tyrosinase	4	6	Others	catechin, phloridzin, quercetin	No	17
2.1.1.B75	flavonoid_7-O-methyltransferase	2	15	Modification	apigenin, kaempferol, luteolin, naringenin, phloretin, quercetin	No	8
1.14.14.89	4”−methoxyisoflavone 2”-hydroxylase	1	24	Modification (addition of hydroxy group)	biochanin A, daidzein, formononetin	Yes	15
2.4.1.5	dextransucrase	35	25	Modification	luteolin, myricetin	Yes	13
2.8.2.1	aryl sulfotransferase	4	32	Modification	apigenin, daidzein, kaempferol, naringenin, phloretin, quercetin, isoquercitrin, rutin, taxifolin	Yes	2
3.3.2.10_3.1.3.76	soluble epoxide hydrolase	4	40	Hydrolysis	chalcone oxides	No	NA
1.10.3.2	laccase	4	50	Others	brenzcatechin, D-catechin, L-epicatechin	No	NA
1.14.12.18	biphenyl 2,3-dioxygenase	17	67	Cleavage	flavone, isoflavone	No	NA
1.13.11.3	protocatechuate 3,4-dioxygenase	130	68	Cleavage	catechin	No	NA
1.14.14.1	unspecific monooxygenase	1	78	Modification (addition of hydroxy group)	apigenin, daidzein, eriodictyol, luteolin, naringenin	Yes	16
3.7.1.4	phloretin hydrolase	21	110	Hydrolysis	dihydrochalcone, phloretin	Yes	map00946
2.4.1.19	cyclomaltodextrin glucanotransferase	28	188	Modification	hesperidin, rutin	No	4
2.4.1.384	NDP-glycosyltransferase	32	235	Modification	apigenin, daidzein, kaempferol, luteolin, naringenin, phloretin, quercetin	No	1
1.8.5.7	glutathionyl-hydroquinone reductase	189	292	Others	2’-(glutathione-S-yl)-quercetin, 2-(glutathione-S-yl)-quercetin	No	NA
2.4.1.4	amylosucrase	1	346	Modification	apigenin, luteolin, phloretin, quercetin, (+)-taxifolin	No	7
2.4.1.7	sucrose phosphorylase	146	517	Modification	(+)-catechin, (-)-epicatechin	Yes	NA
3.2.1.8	endo-1,4-beta-xylanase	129	565	Hydrolysis	catechin	No	NA
2.8.2.22	aryl-sulfate sulfotransferase	221	601	Modification	isoquercetin, quercetin, taxifolin	No	5
1.13.11.24	quercetin 2,3-dioxygenase	760	775	Cleavage	kaempferol, morin, myricetin, quercetin	Yes	18
3.2.1.31	beta-glucuronidase	184	1087	Hydrolysis	apigenin 7,4’-diglucuronide, luteolin triglucuronide, quercetin 3-O-beta-D-glucuronide	No	NA
3.1.6.1	arylsulfatase (type I)	155	1176	Hydrolysis	apigenin-7,4’−disulfate	No	NA
1.1.1.219	dihydroflavonol 4-reductase	173	1303	Modification	eriodictyol, naringenin, (±)-taxifolin	No	19
3.2.1.40	alpha-L-rhamnosidase	609	1378	Hydrolysis (Deglycosylation)	hesperidin, neohesperidin, rutin	Yes	map00946
3.2.1.37	xylan 1,4-beta-xylosidase	634	1558	Hydrolysis (Deglycosylation)	anthocyanin	No	NA
1.3.1.31	2-enoate reductase	137	1926	Cleavage	chalcone	No	NA
2.1.1.104	caffeoyl-CoA O-methyltransferase	184	2316	Modification	eriodictyol, luteolin, myricetin, quercetin	No	10
1.3.1.56	cis-2,3- dihydrobiphenyl-2,3- diol dehydrogenase	19	3013	Cleavage	7-hydroxy-8-methylisoflavone, 7-hydroxyisoflavone	No	NA
3.2.1.20	alpha-glucosidase	1169	3388	Flavonoid interacting	rutin	No	14
3.2.1.21	beta-glucosidase	1327	3625	Hydrolysis (Deglycosylation)	daidzin, genistin, glycitin, naringenin, phloridzin	Yes	map00946
7.6.2.2	ABC-type xenobiotic transporter	485	4606	Flavonoid interacting	phloridzin[side 1], quercetin [side 1]	No	20

The table shows detailed information for 35 potential flavonoid-modifying ECs. Columns include EC number, enzyme names, number of prokaryotic genes obtained from literature, number of UHGG species with homologs for the genes belonging to these ECs, types of modification, flavonoid substrates, previously reported in flavonoid modification in Human Gut, and the potential link of these enzymes with substrates illustrated in Figure 2. See also Supplementary Table S2.

The 6,865 sequences correspond to 39 KEGG orthologs (KO), which map to 47 KEGG pathways, as described in Supplementary Table S3. Among the 35 ECs, 17 ECs that have the potential to act upon substrates mentioned in the flavonoid degradation pathway (map00946) reported in the KEGG database are highlighted in Figure 2. To assess the distribution of potential FMEs in the gut bacterial species, homologs of 6,865 FMEs found in the UHGP catalog were searched for corresponding species representatives in the Unified Human Gastrointestinal Genome catalog v2.0.2 (UHGG) genome metadata. Among the genomes of 4,744 unique gut bacterial species representatives present in the UHGG catalog, we found genomes of 4,606 representative species having homologs for potential FMEs with criteria >30 percent identity (PID), 75% coverage, and 60-bit score (Supplementary Text 1).

### Categories of potential FMEs and distribution of their homologs in the gut bacterial genomes

We categorized the 35 ECs of FMEs into four broad categories based on their putative activities: I) hydrolytic ECs, with the potential to hydrolyze sugar moieties; II) cleavage ECs, with the potential to break down flavonoids and generate other metabolites; III) modifying ECs, with the potential to transfer functional groups to or from flavonoids; and IV) flavonoid-interacting ECs, enzymes with which flavonoids can interact and modulate their activity. The ECs that could not be clearly assigned to any of these categories were grouped under “Others” (Table 1).

Furthermore, we evaluated the prevalence of FMEs attributed to 35 ECs within UHGG genomes. Homologs of FMEs involved in hydrolysis, such as beta-glucosidase, exhibited widespread distribution in genomes, spanning 3,625 species. Homologs for xylan 1,4-beta-xylosidase and alpha-L-rhamnosidase showed intermediate prevalence and were detected in genomes of less than 1,600 species. The homologs for other remaining hydrolases were less abundant and were identified in less than 1,200 species (Table 1). Homologs for FMEs corresponding to the ECs associated with potential cleavage activities were identified across a wide range of species. The number of species harboring homologs for FMEs with potential cleavage activities ranged from two to 3,013 species. For example, homologs of 2-enoate reductase were present in the genomes of 1,926 species, quercetin 2,3-dioxygenase was present in the genomes of 775 species, while homologs for protocatechuate 3,4-dioxygenase, naphthalene 1,2-dioxygenase, and biphenyl 2,3-dioxygenase present in genomes of less than 100 species (Table 1).

Among the FMEs belonging to ECs potentially involved in flavonoid modifications, homologs for caffeoyl-CoA O-methyltransferase were found in genomes of 2,316 species. Likewise, homologs of dihydroflavonol 4-reductase and aryl-sulfate sulfotransferase were found in several genomes spanning 600 to 1,303 species. Homologs of other FMEs in the flavonoid modification category were found to have a lower prevalence and were detected in genomes ranging from 1 to 517 species (Table 1). In the flavonoid interacting enzymes category, homologs of ABC-type xenobiotic transporter sequences were detected with the highest prevalence (genomes of 4,606 species), and alpha-glucosidase was found in the genomes of 3,388 species (Table 1).

### Distribution of biochemically characterized FMEs in the gut bacterial genomes

Since our analysis included both potential and biochemically characterized FMEs, we analyzed the distribution of biochemically characterized FMEs separately, as these have been experimentally reported to be involved in flavonoid metabolism. The biochemically characterized FMEs encompassed sequences involved in various enzymatic activities, including hydrolysis (deglycosylation, derhamnosylation), FMEs specific to flavones/flavonols degradation pathway, equol biosynthesis pathway, and other modifications (hydroxylation and functional group transfer). The distribution of these FMEs in gut bacterial species is summarized in Supplementary Table S4 and Supplementary Text 2.

Homologs for the four key enzymes involved in the flavonol/flavone degradation pathway (Flr, Fcr, Chi, and Phy) were present in the genomes of multiple species, with the number of species carrying homologs for each gene ranging from six to 254. However, the presence of all four genes together within the single bacterial genome was observed in a few species. Homologs of FMEs associated with the equol biosynthesis pathway were also abundant in gut bacterial genomes. For example, homologs for different dihydrodaidzein reductase sequences were found in nine to 1,791 species. However, homologs for daidzein reductase sequences were less prevalent, detected in genomes of only three to 26 species (Supplementary Table S4, Supplementary Text 2).

### Complete enzymatic machinery of flavone/flavonol degradation pathway in few species

To understand the flavonol catabolic capacity of human gut bacteria, we analyzed the presence of homologs for all four key enzymes (Flr, Fcr, Chi, and Phy) in the genomes of the UHGG catalog. Among all species, the genomes of *F. plautii* (MGYG000000099), *Eubacterium_I ramulus (*MGYG000001456.1), *Eubacterium_I sp900546495* (MGYG000000969), and *Eubacterium_I sp900066595* (MGYG000000058) were found to have homologs for all four enzymes, which shows the presence of complete enzymatic machinery for flavones/flavonols degradation pathways in a limited number of species. Here, we also showed that *E. ramulus*, which was previously known to harbor only three genes (Fcr, Chi, and Phy), also has the homologs for the Flr gene (at 59.9 PID with ANU40626.1 and 66.6 PID with ADK16070.1), which is yet uncharacterized.

Interestingly, *Eubacterium_I sp900546495* and *Eubacterium_I sp900066595* harbor the homologs of all four genes (Flr, Fcr, Chi, and Phy), which was previously unreported in these species and indicates their role in flavonoid metabolism. Pairwise average nucleotide identity (ANI) comparison of *Eubacterium_I sp900066595* with *E. ramulus* and *F. plautii* species representative genomes shows OrthoANIu values of 72.24 and 65.44, respectively, indicating that this species is unrelated to these species. When genome abundance was plotted, it was found to be present in all population datasets analyzed in this study, indicating its global presence (Figure 6I). Since among these species, *F. plautii has* been experimentally known to harbor the complete set of genes required for flavonol catabolic activity, we analyzed 638 *F. plautii* genomes present in the UHGG catalog and found that 534 genomes have homologs for all four genes (>90% PID), which suggest that most of the *F. plautii* genomes can break down flavonoids. Genomic coordinates of these four genes in *Eubacterium_I sp900546495, Eubacterium_I sp900066595*, *E. ramulus*, and *F. plautii* are provided in Supplementary Table S5.

### Distinct clustering of genes encoding potential FMEs in healthy Western and non-Western populations

We compared the abundance of genes encoding 6,865 potential FMEs in healthy Western and non-Western populations. A total of 207 genes were found to be differentially abundant in the healthy samples of Western and non-Western populations using Boruta (Supplementary Table S6).

**Figure 3.**
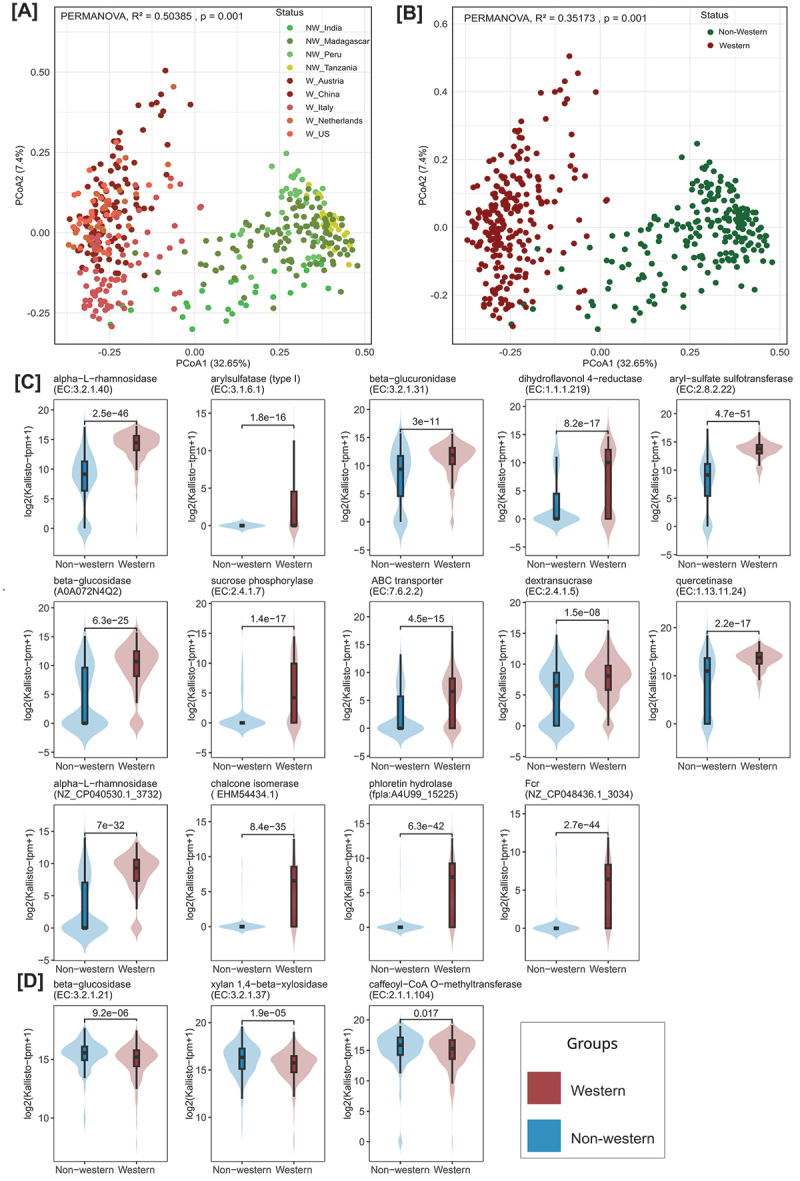
Distinct clustering of genes encoding potential FMEs in healthy Western and non-Western populations. (A, B) Principal coordinate analysis (PCoA) plots considering inter-sample Bray–Curtis distance based on log(tpm + 1) values showing the distribution of Boruta-selected 207 genes in Western and non-Western populations. (C, D) Violin plots with embedded box plots showing differentially abundant potential flavonoid-modifying ECs and genes in Western populations (C), and non-Western populations (D). The Wilcoxon rank-sum test was used to calculate p-values. See also Supplementary Figure S1.

This was further confirmed by Principal Coordinates Analysis (PCoA) analysis using the differentially abundant genes, which showed distinct clustering of the Western and non-Western populations, with the first principal coordinate explaining 32.65% of the variance in the data (Figure 3A,B). The 207 differentially abundant genes were mapped to their corresponding 13 ECs and five biochemically characterized genes (ECs were not available for four biochemically characterized genes). Further analysis indicated that nine ECs, including dihydroflavonol 4-reductase, quercetinase, beta-glucuronidase, dextransucrase, sucrose phosphorylase, aryl-sulfate sulfotransferase, arylsulfatase (type I), alpha-*L*-rhamnosidase, and ABC-type xenobiotic transporter were significantly abundant in Western compared to non-Western populations (Wilcoxon test, *p* < 0.05) (Figure 3C, Supplementary Figure S1A).

Biochemically characterized genes that were highly abundant in Western populations include *F. plautii* encoded *phy*, *chi*, *fcr*, and other genes, including A0A072N4Q2 (Beta-glucosidase) and NZ_CP040530.1_3732 (alpha-L-rhamnosidase) (Figure 3C, Supplementary Figure S1A).

ECs such as beta-glucosidase, caffeoyl-CoA O-methyltransferase, and xylan 1,4-beta-xylosidase showed higher abundance in non-Western populations compared to Western populations (Wilcoxon test, *p* < 0.05) (Figure 3D, Supplementary Figure S1B).

### Distinct clustering of genes encoding potential FMEs in CRC and healthy samples of the Indian population

**Figure 4.**
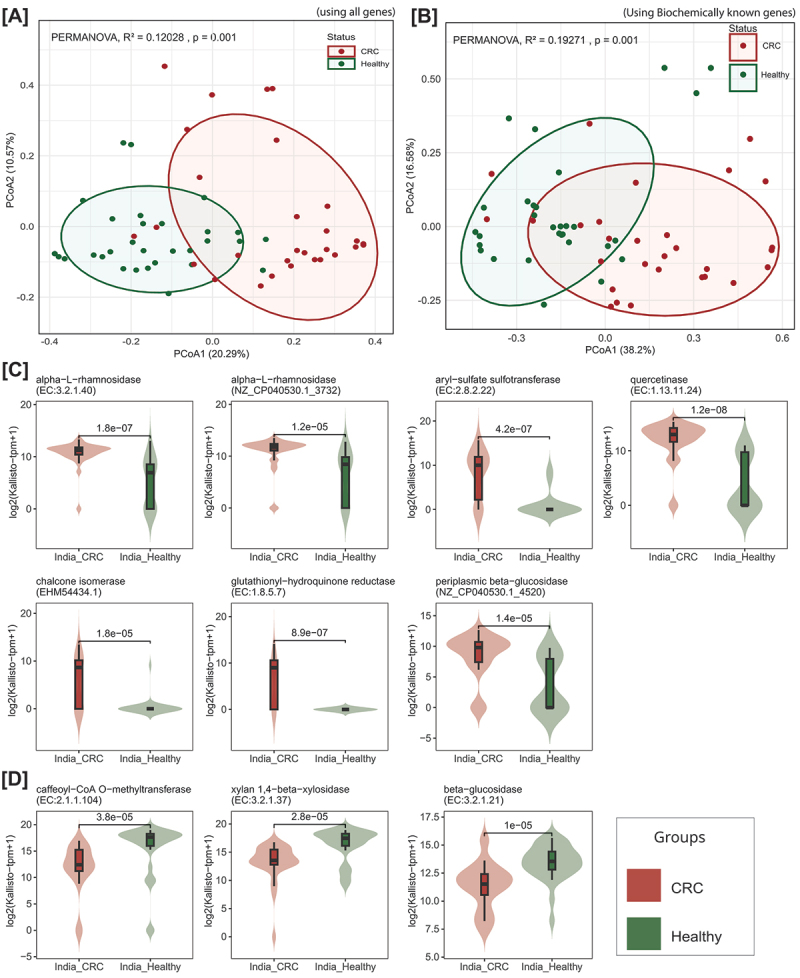
Distinct clustering of genes encoding potential FMEs in CRC and healthy samples of Indian population. (A, B) Principal coordinates analysis (PCoA) plots considering inter-sample Bray–Curtis distance based on log(tpm + 1) values showing the distribution of all potential FMEs (A) and biochemically characterized FMEs (B) in healthy and CRC samples of Indian population. (C, D) Violin plots with embedded box plots showing differentially abundant potential flavonoid-modifying ECs and genes in CRC samples (C) and healthy samples (D). The Wilcoxon rank-sum test was used to calculate pp-values. See also Supplementary Figure S2 and Supplementary Figure S3.

We compared the abundance of genes encoding 6,865 potential FMEs in healthy and CRC samples from different populations. PCoA analysis using inter-sample Bray-Curtis distance showed distinct clustering of CRC and healthy samples in the Indian population (PERMANOVA, R^2^ = 0.12028, *p* = 0.001) (Figure 4A). Furthermore, the analysis of the distribution of 60 biochemically characterized genes in healthy and CRC datasets showed a distinct clustering among Indian CRC and healthy samples (PERMANOVA, R^2^ = 0.19271, *p* = 0.001) (Figure 4B). However, no clear distinction between CRC and healthy samples was observed among Austria (PERMANOVA, R^2^ = 0.01293, *p* = 0.004) and China (PERMANOVA, R^2^ = 0.01701, *p* = 0.004) (Supplementary Figure S2).

Using Boruta, we identified 28 differentially abundant genes that can distinguish between healthy and CRC samples in the Indian population (*p* < 0.05) (Supplementary Table S6). Of these, 25 genes were mapped to seven corresponding ECs, while the remaining three were biochemically characterized genes, with one lacking an assigned EC number.

To assess the differential abundance of these ECs between healthy and CRC groups, we visualized their distributions using boxplots and calculated statistical significance using Wilcoxon’s rank-sum test. This analysis revealed that four ECs, which include quercetinase, glutathionyl-hydroquinone reductase, aryl-sulfate sulfotransferase, and alpha-*L*-rhamnosidase, were significantly enriched in CRC compared to healthy samples in the Indian population. Additionally, three biochemically characterized genes, including alpha-L-rhamnosidase (NZ_CP040530.1_3732), periplasmic beta-glucosidase (bth:BT_1780), and *chi* (EHM54434.1) were highly abundant in CRC compared to healthy samples (Wilcoxon test, *p* < 0.05) (Figure 4C). Furthermore, the ECs, including caffeoyl-CoA O-methyltransferase, beta-glucosidase, and xylan 1,4-beta-xylosidase, were differentially enriched in healthy compared to CRC samples (Wilcoxon test, *p* < 0.05) (Figure 4D).

The 28 genes that showed differential abundance between healthy and CRC samples in the Indian population were not found to be differentially abundant in CRC or healthy groups of the Chinese population. However, in the Austrian dataset, a higher abundance of glutathionyl-hydroquinone reductase, caffeoyl-CoA O-methyltransferase, aryl-sulfate sulfotransferase, xylan 1,4-beta-xylosidase, NZ_CP040530.1_3732, EHM54434.1 was observed in carcinoma samples (but not in adenoma) compared to healthy samples (*p* < 0.05, Wilcoxon test) (Supplementary Figure S3).

### Potential flavonoid modifying ECs were differentially abundant in healthy vs disease and Western vs non-Western populations

**Figure 5.**
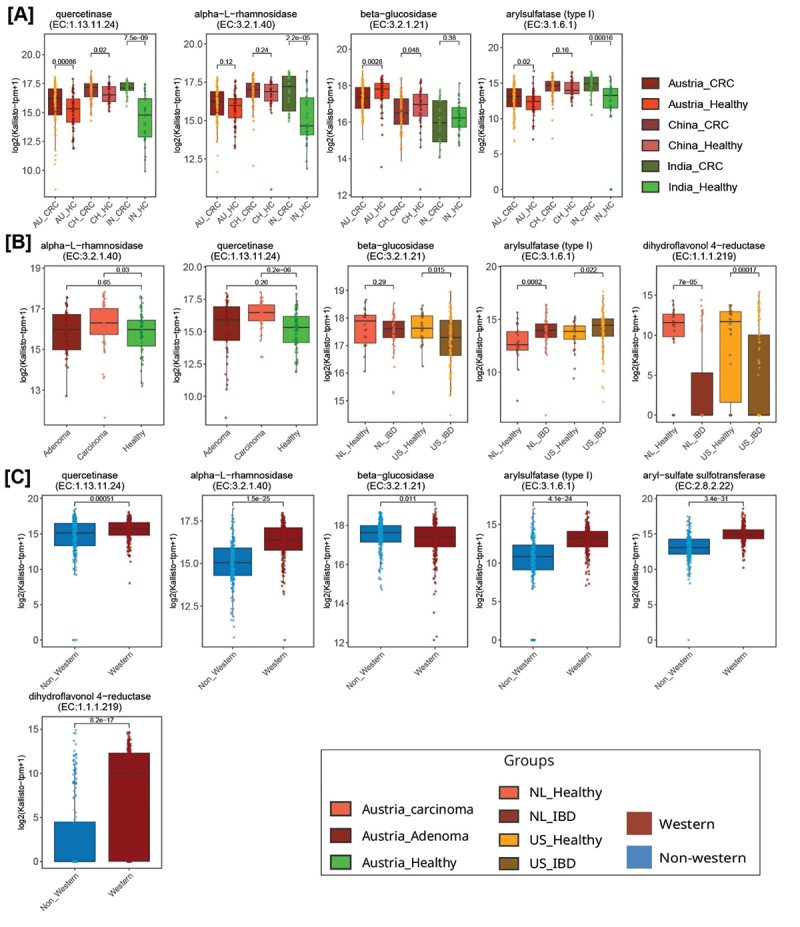
Flavonoid-modifying ECs were differentially abundant in healthy vs. diseased and Western vs. non-Western samples. Boxplots showing differentially abundant potential flavonoid-modifying ECs in healthy vs. CRC samples of Austrian, Chinese, and Indian populations (A). Boxplots showing differentially abundant ECs in healthy vs. adenoma and healthy vs. carcinoma samples of Austrian populations, and healthy vs. IBD samples of the US and Netherlands (NL) populations (B). Boxplots showing differentially abundant ECs in healthy Western vs. healthy non-Western populations (C). Abundance is based on log (tpm + 1) values. The whiskers, bounds of the box, and the line in the middle of the box represent the min-to-max values, 25th–75th percentiles, and median, respectively. The Wilcoxon rank-sum test was used to calculate p-values. See also Supplementary Figure S4 and Supplementary Table S7.

We mapped the abundance of genes encoding 6,865 potential FMEs to their respective ECs and applied Boruta and Wilcoxon tests to identify the ECs that exhibited differential abundance in the three comparisons: CRC vs. healthy, IBD vs. healthy, and Western vs. non-Western across populations. A significantly higher abundance of enzyme quercetinase (EC:1.13.11.24) was observed in CRC samples from India, China, and Austria compared to healthy samples of their corresponding populations (*p* < 0.05) (Figures 5A and 4C, Supplementary Figure S4A, S4F). The enzyme alpha-L-rhamnosidase (EC:3.2.1.40), which hydrolyzes terminal L-rhamnose residues from glycosides, showed significantly higher abundance in CRC samples from India and carcinoma samples from Austria, compared to their corresponding healthy groups (*p* < 0.05) (Figures 5A,B and 4C, Supplementary Figure S4B, S4G). It also showed a higher abundance in CRC compared to healthy samples from China (Figure 5A, Supplementary Figure S4B). Both the enzymes were significantly highly abundant in Western compared to non-Western populations (*p* < 0.01) (Figures 5C and 3C, Supplementary Figure S4K, S4L).

The abundance of beta-glucosidase (EC:3.2.1.21) was significantly higher in healthy samples of Austria and China compared to CRC samples (*p* < 0.05) (Figure 5A, Supplementary Figure S4C). The enzyme was also differentially abundant in healthy samples of the US (*p* = 0.015) and Netherlands (*p* > 0.05) compared to IBD samples of the respective populations (Figure 5B, Supplementary Figure S4H). Beta-glucosidases exhibit broad substrate specificity and catalyze the hydrolysis of beta-glycosidic linkage in various dietary components.

The enzyme arylsulfatase (type I) (EC:3.1.6.1) exhibited significantly higher abundance in CRC samples of India and Austria compared to healthy samples of corresponding populations (*p* < 0.05) (Figure 5A, Supplementary Figure S4D). It also showed significantly higher abundance in IBD compared to healthy samples from the US and Netherlands populations (*p* < 0.05) (Figure 5B, Supplementary Figure S4I). This enzyme was significantly enriched in Western compared to non-Western populations (*p* < 0.01) (Figure 5C, Supplementary Figure S4N). Notably, arylsulfatase (type I) catalyzes the hydrolysis of aryl sulfate ester bonds to release a free sulfonate and has the potential to act upon apigenin-7,4’-disulfate.^44^

Aryl-sulfate sulfotransferase (EC:2.8.2.22) showed significantly higher abundance in Western compared to non-Western populations (Figure 5C, Supplementary Figure S4O). Enzyme dihydroflavonol 4-reductase (EC:1.1.1.219) was significantly highly abundant in healthy samples from the US and the Netherlands compared to corresponding IBD samples and in Western compared to non-Western populations (*p* < 0.01) (Figure 5B,C, Supplementary Figure S4J, S4M). This enzyme is primarily known for its role in the biosynthesis of anthocyanidins and other flavonoids in plants, where it catalyzes the reduction of dihydroflavonols. Its presence and functional roles in bacteria are less documented.^45,46^ The p-values for differentially abundant ECs are provided in Supplementary Table S7.

### Biochemically characterized FMEs were differentially abundant in healthy vs. disease and Western vs non-Western populations

**Figure 6.**
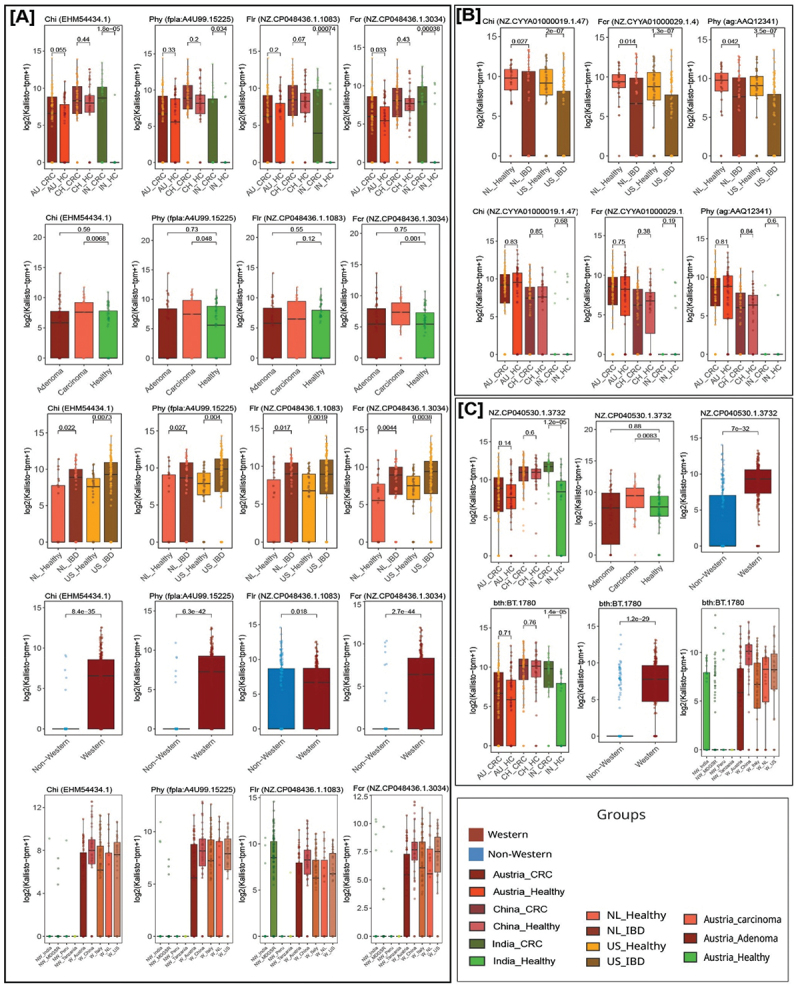
Biochemically characterized FMEs were differentially abundant in healthy vs. disease and Western vs. non-Western populations. Boxplots based on abundance (log(tpm + 1)) showing differentially abundant flavonoid-metabolizing genes encoding *chi* (EHM54434.1), *phy* (fpla:A4U99_15225), *flr* (NZ_CP048436.1_1083), and *fcr* (NZ_CP048436.1_3034) in healthy vs. CRC samples of Austrian, Chinese, and Indian populations, healthy vs. Adenoma and healthy vs. carcinoma samples of Austrian population, healthy vs. IBD samples of US and Netherlands (NL) populations, and healthy Western vs. healthy non-Western populations (A). Boxplots showing differentially abundant flavonoid-metabolizing genes encoding *chi* (NZ_CYYA01000019.1_47), *fcr* (NZ_CYYA01000029.1_4), and *phy* (ag:AAQ12341) in healthy vs. IBD samples of US and Netherlands populations and boxplots showing lower abundance of these genes in both healthy and CRC samples from Indian compared to other populations (B). Boxplots showing differentially abundant periplasmic beta-glucosidase (bth:BT_1780) and alpha-L-rhamnosidase (NZ_CP040530.1_3732) in healthy vs. CRC samples of Austrian, Chinese, and Indian populations; healthy vs. adenoma and healthy vs. carcinoma samples from Austrian populations; and healthy Western vs. healthy non-Western populations (C). The whiskers, bounds of the box, and the line in the middle of the box represent the minimum to maximum values, 25th to 75th percentiles, and median, respectively. The Wilcoxon rank-sum test was used to calculate p-values. See also Supplementary Figure S5 and Supplementary Table S7.

The differential abundance of biochemically characterized FMEs was examined using Wilcoxon’s test among CRC vs. healthy, IBD vs. healthy, and healthy Western vs. non-Western groups. A significantly higher abundance of the *chi* gene (EHM54434.1, K26177) of *F. plautii* was observed in CRC samples of India and carcinoma samples of Austria compared to healthy samples of the corresponding cohorts (*p* < 0.01). Additionally, this gene exhibited a higher abundance in CRC samples from China, although the differences were not statistically significant (*p* = 0.44). A significantly higher abundance of this gene was also observed in the US and Netherlands IBD samples compared to the corresponding healthy samples (*p* < 0.05) (Figures 4C, 6A, Supplementary Figure S5A).

A similar abundance pattern was observed for *fcr* (NZ_CP048436.1_3034, K26178) and *phy* (fpla:A4U99_15225, K22906) genes. These genes were differentially abundant in the IBD samples of US and the Netherlands, and CRC and carcinoma samples of India and Austria samples compared to the healthy samples of the respective populations (*p* < 0.05). Moreover, *flr* gene (NZ_CP048436.1_1083) (K26176) of *F. plautii* showed significantly higher abundance in IBD samples from the US and the Netherlands compared to the corresponding healthy groups. This gene was significantly enriched in CRC compared to healthy samples of the Indian dataset (*p* < 0.05) (Figure 6A, Supplementary Figure S5A).

**Figure 7.**
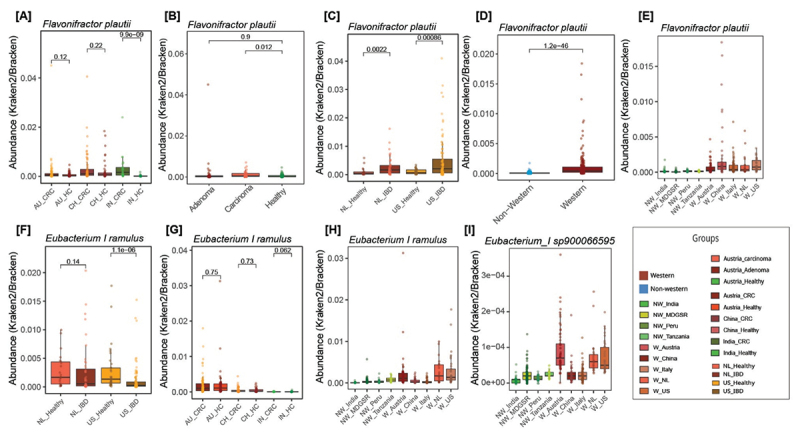
Relative abundance of *F. plautii, Eubacterium_I ramulus, and Eubacterium_I sp900066595* in various populations. Boxplots showing the relative abundance (obtained from classification of reads using Kraken2/Bracken) of *F. plautii* species in healthy vs. CRC samples from Austria, China, and India; healthy vs. adenoma and healthy vs. carcinoma samples from Austria; healthy vs. IBD samples from the US and Netherlands; and healthy Western vs. healthy non-Western populations (A-E). Boxplots showing the abundance of *Eubacterium_i ramulus* species in healthy vs. IBD samples from the US and Netherlands; healthy vs. CRC samples from India; and healthy Western vs. healthy non-Western populations (F-H). Boxplots showing the abundance of *Eubacterium_i sp900066595* in healthy Western vs. healthy non-Western populations (I). The whiskers, bounds of the box, and the line in the middle of the box represent the minimum to maximum values, 25th to 75th percentiles, and median, respectively. The Wilcoxon rank-sum test was used to calculate p-values.

Notably, all four genes (K22906, K26176, K26177, K26178) were significantly enriched in Western populations compared to non-Western populations (*p* < 0.05) (Figures 6A and 3C, Supplementary Figure S5A). To further validate these observations, the genome abundance of *F. plautii* in various population datasets was analyzed. The results revealed a significantly higher abundance of *F. plautii* in Indian CRC and Austrian carcinoma compared to healthy samples of the respective populations (*p* < 0.05) (Figure 7A,B). Moreover, a significantly higher abundance of *F. plautii* was observed in IBD samples of the US and the Netherlands compared to healthy samples (*p* < 0.01) (Figure 7C). A higher enrichment of *F. plautii* was also observed in Western compared to non-Western populations (*p* < 0.01) (Figure 7D,E).

Three genes encoding Chi (NZ_CYYA01000019.1_47) (K26177), Phy ag:AAQ12341 (K22906), and Fcr (NZ_CYYA01000029.1_4) (K26178) enzymes were differentially abundant in healthy compared to IBD samples of US and Netherlands populations (*p* < 0.05) (Figure 6B, Supplementary Figure S5B). These three genes were found to be from the *E. ramulus* genome. Upon analyzing the abundance of the *E. ramulus* genome in different population datasets, it showed higher abundance in healthy samples of the US and the Netherlands compared to IBD samples of corresponding groups (Figure 7F). Surprisingly, this bacterium was abundant in Western and non-Western populations examined in this study except India, where the abundance of these three enzymes and the *E. ramulus* genome was negligible in Indian healthy and CRC samples (present in less than 10 percent of the total samples) (Figure 7G,H).

Further, our analysis indicated that enzyme alpha-L-rhamnosidase (NZ_CP040530.1_3732) encoded by *Bacteroides thetaiotaomicron* VPI-5482 was differentially abundant in India, CRC and Austrian carcinoma samples compared to their corresponding healthy groups (*p* < 0.01) (Figures 4C and 6C, Supplementary Figure S5C). The flavonoid-modifying potential of alpha-L-rhamnosidase has been validated experimentally and the enzyme hydrolyzes alpha-1,2 glycosidic bond of rutinosylated flavonoids.^8^ The *bth:BT_1780* (K05349) gene showed significantly higher differential abundance in Indian CRC compared to healthy samples (*p* = 1.4e − 05) (Figure 4C). Also, a higher abundance (not significant) of this gene was observed in CRC samples from Austrian and Chinese populations (*p* > 0.05). Both NZ_CP040530.1_3732 and bth:BT_1780 genes were significantly enriched in Western compared to non-Western populations (*p* < 0.01) (Figure 6C).

We further observed that genes associated with the equol biosynthesis pathway were present in a limited number of samples across the healthy populations considered in this study (except for some genes that were present in many individuals of a few populations) (Supplementary Table S8). All the genes analyzed above are associated with the flavonoid degradation pathway (map00946) reported in the KEGG database shown in Figure 2A,B. The p-values for differentially abundant genes are provided in Supplementary Table S7.

## Discussion

The gut microbiome plays a crucial role in metabolizing dietary flavonoids, influencing their absorption and health benefits. While over 10,000 flavonoids have been reported in the literature and some studies have identified specific bacterial species and enzymes involved, the understanding of their metabolism by gut microbiome remains limited. Existing databases provide general information on flavonoids, such as chemical structures and physicochemical properties,^47,48^ but no comprehensive database focuses on flavonoid-metabolizing enzymes from human gut bacteria.

Thus, the first step in this work involved the construction of a comprehensive database containing 6,865 FMEs from bacteria, including 60 biochemically characterized FMEs from human gut bacteria from published literature, and potential FMEs belonging to 35 ECs obtained from biochemical databases (BRENDA and KEGG). This helped in the analysis of the abundance of FMEs in healthy individuals from populations with distinct dietary habits (Western and non-Western) and those with IBD and CRC and improved our understanding of flavonoid metabolism in the human gut.

Importantly, we found 24 ECs (out of total 35) to be associated with microbial flavonoid metabolism, which was not known earlier, and indicate the presence of a large number of yet uncharacterized enzymatic reactions within the gut microbiome for flavonoid modifications. The ECs such as arylsulfatase (type I), aryl-sulfate sulfotransferase, glutathionyl-hydroquinone reductase, caffeoyl-CoA O-methyltransferase, xylan 1,4-beta-xylosidase were widely distributed in gut bacterial genomes and may contribute to flavonoid modifications that are yet unreported. For example, arylsulfatase (type I) catalyzes the hydrolysis of aryl sulfate ester bonds, which was not previously associated with flavonoid metabolism in the human gut. Since it has the potential to act upon apigenin-7,4’-disulfate, its possible role in gut flavonoid modification is suggested.^44^ The higher abundance of arylsulfatase (type I) in diseases like IBD and CRC, as well as in healthy Western populations, further highlights its association with these conditions.

Similarly, aryl-sulfate sulfotransferase catalyzes the transfer of a sulfate group from a phenol sulfate ester to other phenolic compounds, including quercetin, increasing its water solubility and resulting in a less absorbable form.^49^ It is suggested that intestinal flora-harboring aryl-sulfate sulfotransferase is involved in the metabolism and detoxification of phenolic compounds.^50,51^ Other ECs, such as alternansucrase, estrone sulfotransferase, naphthalene 1,2-dioxygenase, catechol *O*-methyltransferase, tyrosinase, 2-heptyl-1-hydroxyquinolin-4(1H)-one methyltransferase, and flavonoid 7-*O*-methyltransferase, showed limited distribution in gut bacterial genomes. Additionally, the number of bacterial FMEs obtained from biochemical databases for these ECs was also low, which suggests that such modification reactions are not commonly present in the human gut.

Our findings revealed diverse bacterial species harboring homologs for potential FMEs, including many not previously reported. The identification of 8,74,962 homologs across 2,88,942 UHGG genomes, representing 4,722 species, indicates that flavonoid-modifying capabilities are widespread in the gut microbiome. In addition to the well-characterized flavonoid metabolizing species like *F. plautii* and *E. ramulus*, the presence of FME homologs in species not previously associated with flavonoid metabolism highlights the wide presence of these capabilities in gut microbial species. For example, the presence of homologs for diverse FMEs among members of genera like *Faecalibacterium*, *Bifidobacterium*, and *Bacteroides*, commonly regarded as beneficial gut commensals, expands the potential microbial repertoire involved in flavonoid modification. Thus, the findings in this study suggest that flavonoid metabolism may be more taxonomically widespread than previously known and provide leads for further experimental validations of such activities in lesser-studied gut species.

While certain species and ECs may play pivotal roles in flavonoid metabolism, others might be involved in some functions that are associated with flavonoid metabolism but are primarily a part of other metabolic pathways. For example, the high prevalence of ABC xenobiotic transporter (category IV) homologs in 4,606 species likely reflects their essential role in exporting diverse substances, including drugs, toxins, and xenobiotics. This ability might be helpful in survival and adaptation in the complex gut environment. Since flavonoids can potentially inhibit these transporters, their interaction holds importance in the gut environment.^52^ Furthermore, our results indicate that of the two major steps in flavonoid metabolism, glycone to aglycone conversion, and aromatic ring breakage or modification, FMEs associated with the first step are widespread among gut microbes, while the latter is restricted to a narrow range of species.

As shown in Figure 2, four key enzymes (Flr, Chi, Phy, and Fcr) participate in flavones/flavonols degradation pathway, carrying out the degradation of flavonoids into monophenolic acids such as 3-(4-hydroxyphenyl) propionic acid (HPPA) and phloroglucinol (PG), which have well-documented protective effects against cellular oxidative stress.^20–22^ Earlier, the presence of only three (*chi, phy*, and *fcr*) out of the four genes was known in *Eubacterium_I ramulus*. In this work, we identified the presence of a potential *flr* gene that remains uncharacterized to date. Thus, among all the bacteria that were identified to have these genes, homologs for all four genes were identified in a few species, including *F. plautii*, *Eubacterium_I ramulus*, *Eubacterium_I sp90054649*, and *Eubacterium_I sp900066595*. The limited distribution of these enzymes reflects the complexity and specificity of this pathway, which remains confined to a few gut species. These enzymes, such as dioxygenases, are specialized and rare among gut microbes, as they perform complex oxidative reactions to cleave the stable aromatic structures of flavonoids. Furthermore, the metabolic pathways involved in aromatic ring degradation are energy-intensive and are typically present in bacteria that have evolved in environments where flavonoid degradation provides a competitive advantage, explaining the narrow distribution of this function within the gut microbiota.

Another highlight of this work was the identification of *Eubacterium_I sp900066595* as a new *Eubacterium* species that contains homologs of all four key genes of the flavonoid metabolism pathway. The widespread distribution of this bacterium across various populations analyzed in this study makes it tempting to speculate the prominent play of this species in flavonoid metabolism in the human gut. However, these results need further experimental validation.

Furthermore, the differential abundance of *F. plautii* genes (*flr, chi, phy, and fcr*) in Western compared to non-Western populations suggests that diverse dietary habits may play a role in shaping the abundance of flavonoid-modifying bacterial enzymes in the gut microbiome. Interestingly, the higher abundance of these genes was also observed in IBD and CRC compared to corresponding healthy samples. As suggested by Gupta et al., degradation of flavonoids by *F. plautii* in CRC may lead to lower availability of flavonoids.^53^

Our analysis also revealed a higher abundance of *F. plautii* species in healthy Western populations and IBD and CRC datasets. Recent studies have linked *F. plautii* to less healthy dietary patterns, showing a negative association with plant-based diets and a positive association with less healthy dietary choices, such as sugar-sweetened beverages and Western-style fast foods.^54–56^ Beyond diet, other host factors may contribute to its enrichment in disease conditions. For example, the enrichment of *F. plautii* in IBD patients was attributed to elevated levels of mucosal IgG in their intestinal tracts, and it was found that *F. plautii* was preferentially bound by IgG in IBD patients.^57^ Additionally, its ability to occupy various niches, compete for resources, and interact with other species in the gut microbiome may favor its abundance in the dysbiotic gut environment.^58^

With these observations, the higher abundance of *F. plautii* in individuals with IBD, CRC, and Western dietary patterns raises intriguing questions about its role in gut health. While few studies suggested its potential as a probiotic in specific cases, most of the other studies have highlighted its pathogenic potential.^57,59–62^ This duality suggests that the abundance of *F. plautii* in the gut could have both beneficial and harmful effects on health. Thus, despite its capacity to metabolize dietary flavonoids, its enrichment in IBD, CRC, and healthy Western populations warrants further investigation to understand its precise role in human health.^53,57,62,63^

In addition to *F. plautii*, *E. ramulus* has been extensively investigated for its ability to degrade flavonoids through the three key genes encoding Fcr, Chi, and Phy in this species.^64^ These genes were differentially abundant in healthy compared to the IBD samples from the US and Netherlands populations.^22,65^ These results highlight the importance of these genes in flavonoid degradation in the healthy gut. Interestingly, our analysis revealed a low abundance of this species in both Indian healthy and CRC samples, contrasting with previous reports that indicated a higher prevalence of this species in the human gut.^66^ Given the low abundance of both *E. ramulus* and *F. plautii*, which are significant contributors to flavonoid degradation in the healthy Indian gut, there is a possibility of yet unknown role or presence of other bacteria in the flavones/flavonols degradation pathways.

Another key observation was the higher abundance of periplasmic beta-glucosidase encoding gene (bth:BT_1780) of the *Bacteroides thetaiotaomicron VPI-5482* in Indian CRC samples and healthy samples of Western populations. *Bacteroides thetaiotaomicron VPI-5482* possesses numerous genes that participate in carbohydrate metabolism, including multiple beta-glucosidase genes. The enzyme encoded by bth:BT_1780 specifically exhibits hydrolyzing activity toward isoflavone glycosides. Unlike other beta-glucosidases, it does not hydrolyze xylo-oligosaccharides.^67^ The breakdown of isoflavone glycosides by this gut bacteria enhances the bioavailability and increases their absorption by the host, which has health-promoting effects.^68,69^ Enrichment of this enzyme in CRC patients and Western populations may favor the biotransformation of isoflavones and thus plays a beneficial role. The genes involved in the equol biosynthesis pathway, including daidzein reductase, dihydrodaidzein racemase, dihydrodaidzein reductase, and tetrahydrodaidzein reductase, were present in fewer samples of analyzed population datasets. Previous studies have reported that the frequency of equol producers varies from 30% to 60% of individuals from Western and non-Western populations, respectively.^68,70,71^

Interestingly, the gut microbiome of CRC patients displayed a higher abundance of alpha-L-rhamnosidase, which indicates a higher derhamnosylation potential compared to healthy individuals. Quercetinase, which cleaves C–C bonds in flavonols, has been studied primarily in plant-associated fungi but is also present in bacteria and was first identified in *Bacillus subtilis*.^72^ The Pirin protein YhhW in *E. coli* also exhibits quercetinase activity.^73^ It has been suggested that quercetinase may play a detoxification role in bacteria, as flavonols within bacterial cells can have deleterious effects.^74^ Modifying quercetin by quercetinase, followed by the action of gut esterase on the intermediary product, can result in the formation of 2,4,6-trihydroxybenzoic acid (2,4,6-THBA) and 3,4-dihydroxybenzoic acid (3,4-DHBA).^59^ Notably, 2,4,6-THBA is a CDK inhibitor and an anti-proliferative agent.^75^ A significantly higher abundance of quercetinase in CRC compared to healthy samples across cohorts and Western compared to non-Western populations suggests quercetinase may degrade flavonoids through an alternate reaction (rn:R02156) in CRC and Western gut microbiomes.^76^

In summary, our study uncovers novel gut bacterial enzymes and species potentially involved in flavonoid metabolism, which highlights the complexity of flavonoid metabolism and offers valuable clues for future research. It offers insights into the variations in abundance of FMEs across diverse populations with varying dietary patterns and diseases, including IBD and CRC. The abundance of *F. plautii* encoding Flr, Chi, Phy, and Fcr in both IBD and CRC samples suggests their potential association with these diseases. Moreover, the higher prevalence of *F. plautii* enzymes in Western compared to non-Western populations hints at dietary habits influencing the abundance of these FMEs. Additionally, the higher abundance of *E. ramulus* genes (*Fcr, Chi, and Phy*) in healthy compared to IBD samples highlights the possible association of *E. ramulus* with healthy gut microbiome. The identification of the Flr homolog in *E. ramulus*, as well as homologs for Flr, Chi, Phy, and Fcr in *Eubacterium_I sp900546495* and *Eubacterium_I sp900066595* species, highlights the yet unexplored flavonoid metabolism in the human gut. Higher abundance of quercetinase and alpha-L-rhamnosidase in CRC compared to healthy samples suggests a potential association of these enzymes with CRC. The human gut-microbiome associated flavonoid-modifying enzymes and species, their roles, and distribution with respect to health and disease in various populations reported in this study provide clues for further examination in larger datasets and for experimental validations that hold promise for therapeutic interventions and usage as diagnostic markers in various dysbiosis conditions. A website providing detailed information on the FMEs analyzed in this study is freely accessible at https://metabiosys.iiserb.ac.in/pfme.

Our study reported both biochemically characterized flavonoid-metabolizing enzymes from previous literature and identified novel ECs with the potential to act upon flavonoids, with their homologs in the human gut microbiome. While these homologs may act upon flavonoids, many of these could also be potentially involved in other metabolic pathways. Therefore, experimental validation will further confirm their specific roles in flavonoid metabolism.

Additionally, some experimentally characterized enzymes lack assigned EC numbers, reflecting a gap between experimental characterization and formal annotation of uncharacterized genes in human metagenomic datasets. This is one of the key challenges in the identification of potential genes that could be associated with flavonoid metabolism and their functional annotations, which are limited by the representation of annotated functional genes in the reference databases.^77^ Thus, the growing knowledge of EC assignments in metagenomic and biochemical studies, along with the integration of experimental approaches and computational homology-based predictions, will enhance our understanding of flavonoid-modifying enzymes in the gut microbiome.

## Materials and methods

### Retrieval of potential FMEs from the BRENDA and KEGG databases

Potential FMEs were retrieved from the literature using multiple approaches. A list of 114 flavonoid-related keywords was obtained from Ivey et al.’s study (Supplementary text 3). The “quick search” option of the BRENDA database was used^44^ to search these keywords, resulting in the retrieval of 232 EC numbers representing enzymes capable of utilizing flavonoids as substrates. A total of 3,37,665 protein sequences associated with these ECs were retrieved. Similarly, these keywords were searched in the KEGG database using the KEGGREST function available in the KEGG API and obtained 42 EC numbers corresponding to flavonoid modifying enzymes along with 55,110 protein sequences belonging to these ECs.^78^ Combining the protein sequences obtained from both the KEGG and BRENDA databases resulted in a total of 3,92,775 potential flavonoid modifying proteins, which constitute the “FME database” of potential flavonoid modifying proteins found in prokaryotes and eukaryotes.

### Retrieval of biochemically characterized gut bacterial FMEs from literature

The existing literature was reviewed to identify biochemically characterized gut bacterial proteins involved in flavonoid metabolism, resulting in the inclusion of 60 additional protein sequences in the FME database^79,80^ (Supplementary Table S1).

### Database of potential FMEs of prokaryotic origin

The sequence IDs in the FME database were compared with eukaryotic KEGG organism codes available in the KEGG database to identify and eliminate sequences of eukaryotic origin, thereby creating a dataset consisting exclusively of prokaryotic sequences. Subsequently, the prokaryotic sequences in the FME database were clustered using CD-HIT version 4.8.1^81^ with a 100% sequence identity threshold. The longest sequence from each cluster was considered as the representative sequence to construct a set of non-redundant protein sequences. As a result, the updated FME database comprised 30,601 protein sequences belonging to 95 unique EC numbers specific to prokaryotes (Supplementary Table S9).

### Database of potential FMEs in human gut bacterial genomes of the UHGG catalog

The identification of potential FMEs in human gut bacteria was achieved by retrieving 95,303,635 protein sequences (clustered at 100% identity) of human gut bacterial genomes from the UHGP catalog.^82^ The UHGP database, derived from the UHGG, which contains 2,89,232 bacterial genomes representing 4,744 unique species clusters, comprising both isolate and metagenome-assembled genomes (MAGs).^82^ Subsequently, the UHGP sequences were mapped against the database of 30,601 bacterial protein sequences identified in the previous step using Diamond software v2.0.15.153.^83^

At a minimum percent identity threshold of 30%, coverage of 75%, and a 60-bit score, 6,865 out of 30,601 bacterial protein sequences were identified as having homologs in the UHGP catalog. These 6,865 protein sequences belong to 35 EC numbers (except 41 biochemically characterized FMEs for which EC numbers were not available) (Supplementary Table S10). The KEGG ortholog IDs of the 6,865 protein sequences and corresponding pathway maps were identified. The entire process for constructing the database of potential FMEs present in gut-residing bacteria is summarized in Figure 1. The basis for criteria selection is discussed in Supplementary Text 4. To assess the distribution of FMEs in the human gut bacterial genomes, homologs of 6,865 FMEs found in the UHGP catalog were investigated in the UHGG metadata that contains detailed information for each genome and corresponding representative species. A web-based interface was developed to provide users with access to key details of biochemically characterized FMEs and 35 potential flavonoid-modifying ECs.

### Obtaining publicly available human gut metagenomic data

**Figure 8.**
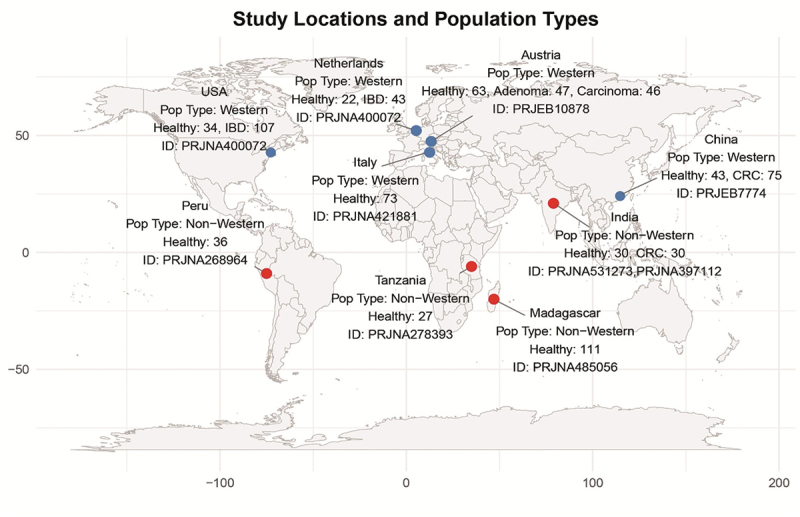
Geographic distribution of metagenomic datasets included in this study.

We downloaded gene sequences of 6,865 potential FMEs and investigated the abundance of these genes in the gut microbiome of 787 individuals with different dietary habits and disease conditions Figure 8. This includes samples from healthy individuals and patients diagnosed with CRC and IBD, including ulcerative colitis (UC) and Crohn’s disease (CD).

Publicly available metagenomic datasets were downloaded from the NCBI Sequence Read Archive (SRA). Healthy datasets include samples of 111 healthy individuals from Madagascar,^84^ 27 healthy individuals from Tanzania,^85^ 36 healthy individuals from Peru,^86^ 73 healthy individuals from Italy,^87^ 34 healthy individuals from the US, 22 healthy individuals from the Netherlands,^88^ 30 healthy individuals from India,^53^ 43 healthy individuals from China^89^ and 63 healthy individuals from Austria.^90^ Healthy population data were further characterized as Western (US, Netherlands, Spain, Italy, China, Austria) and non-Western (India, Madagascar, Tanzania, Peru) based on their dietary variations.^91^ The Chinese dataset (collected from Hong Kong) was classified as Western based on the findings of the recent studies that reported the gut microbial compositions of urban Chinese populations with westernized dietary patterns exhibit high similarity to the gut microbiome composition of Western countries.^92,93^ Disease datasets include samples from 107 IBD (54 CD and 53 UC) patients from the US, 43 IBD (20 CD, 23 UC) patients from the Netherlands,^88^ 30 CRC patients from India,^53^ 75 CRC patients from China,^89^ and 47 advanced Adenoma, and 46 carcinoma patients from Austria.^90^

### Data preprocessing and quality control

Raw FASTQ files were downloaded from the SRA (accession codes are provided in the data availability section). The raw reads were trimmed to remove low-quality bases and adapter sequences using Trimmomatic version 0.38.^94^ The resultant high-quality reads were filtered to remove potential host-origin reads (human DNA contamination) using Bmtagger version 3.101 by aligning high-quality reads against the GRCh38 human reference genome.^95^ The read statistics for each dataset are provided in Supplementary Table S11.

### Calculating gene abundance in metagenomic datasets

The abundance of genes was estimated using kallisto version 0.46.1.^96^ Nucleotide sequences of 6,865 FMEs were downloaded, and the index file of gene sequences was created using the index function in kallisto. High quality reads were then pseudo-aligned using the quant function in kallisto with default parameters. Gene abundance was quantified using transcripts per million (tpm) values. Subsequently, the abundance of ECs was calculated from gene abundance data. To validate the results obtained from kallisto, a confirmatory analysis was carried out using SOAPaligner version 2.21,^97^ which involved the alignment of high-quality reads against index files of the same set of genes generated using SOAPaligner. Similar results were obtained from both tools, and the results from SOAPaligner are provided in Supplementary Figure S4 and S5.

### Identifying differentially abundant genes

We analyzed the abundance of genes encoding potential FMEs in samples from Western and non-Western healthy populations and different health conditions (healthy vs. CRC, healthy vs. IBD). After adding pseudo-counts, gene abundance was log-transformed (log_2_(tpm +1)). Using (log_2_(tpm +1)), the BrayCurtis distance matrix was calculated. PCoA was carried out using QIIME2 version 2023.7.0.^98^ Inter sample variation was assessed using the PERMANOVA test using the adonis function in QIIME2. Statistical analysis was performed using RStudio with R version 4.3.2. Boruta package in R was utilized to identify the differentially abundant genes between different groups (healthy Western vs. non-Western, healthy vs. CRC, and healthy vs. IBD).^99^ The abundance of genes selected based on Boruta analysis (*p* = 0.01) was plotted as boxplots using the ggplot function in R. The significance of differential abundance between two groups was tested using a two-sided Wilcoxon rank-sum test. Kruskal–Wallis’ test was applied to find significant differences between multiple groups.

### Calculating the differential abundance of bacterial species in metagenomic datasets

The abundance of selected bacterial species involved in flavonoid metabolism in the gut was evaluated across different population datasets. Genome sequences of species representatives from the UHGG catalog v2.0.2 were retrieved.^82^ The Kraken2 custom database was created using these sequences. Taxonomic classification of metagenomics reads performed with Kraken2 against the custom database of UHGG species representatives. Bracken was used to calculate relative abundance from the Kraken2 profile. The relative abundance of selected species was plotted as box plots using the ggplot function in R. The significance of differential abundance was evaluated using a two-sided Wilcoxon rank-sum test.

### Genome analysis

To identify *F. plautii* genomes containing all four key enzymes (Flr, Fcr, Chi, and Phy), the 638 *F. plautii* genomes available in the UHGG catalog v2.0.2 (20^th^ May 2024) were retrieved, and gene prediction was carried out using Prodigal v2.6.3.^100^ Predicted proteins were aligned against the FMEs protein database using Diamond software v2.0.15.153. At a minimum percent identity threshold of 90% and e-value of 10^−6^, the presence of homologs for four key genes was identified. We also extracted the genomic coordinates of the homologs for flr, fcr, chi, and phy enzymes from the GFF files of genomes containing all four enzymes. These genomes include *F. plautii, Eubacterium_I ramulus, Eubacterium_I sp900546495, and Eubacterium_I sp900066595*. Pairwise average nucleotide identity was calculated using orthoANI, considering orthologous genome fragments.^101^

## Supplementary Material

Supplementary_Table_6.xlsx

Supplementary_Table_11.xlsx

Supplementary_Table_9.xlsx

Supplementary_Table_10.xlsx

Supplementary_Table_3.xlsx

Supplementary_text_150525.docx

Supplementary_table_2.xlsx

Supplementary_table_8.xlsx

Supplementary_Table_1.xlsx

Supplementary_Table_5.xlsx

Supplementary_table_7.xlsx

Supplementary_table_4.xlsx

## Data Availability

Metagenomic data used in this paper are publicly available under the following BioProject IDs: PRJNA400072, PRJNA421881, PRJNA531273, PRJEB7774, PRJEB10878, PRJNA268964, PRJNA278393, PRJNA485056, and PRJNA397112.
