# Supplementary material for: Landscape of flavonoid metabolism in human gut microbiome and its association with health and disease
Source: Gut Microbes Rep. 2025 Jun 25;2(1):2520788. doi: 10.1080/29933935.2025.2520788 (PMC12940152; doi:10.1080/29933935.2025.2520788)
Supplement: Supplementary_text_150525.docx [file KGMR_A_2520788_SM8845.docx]

**Supplementary text 1**

**Homologs for potential FMEs present in genomes of several UHGG species**

Upon analyzing the distribution of 8,74,962 homologs in the gut bacterial genomes present in the UHGG catalog, we observed that a wide range of bacteria possess homologs for potential FMEs, including species previously not reported to be involved in flavonoid metabolism. We found that 2,88,942 genomes that represent 4,722 corresponding species representatives were found with at least one homolog for potential FMEs. Some selected cases of bacterial species with homologs for potential FMEs with 90 percent identity cutoff are mentioned in **Supplementary Table 12**. For example, *Phocaeicola dorei (Bacteroides dorei)* is a Gram-negative bacterium contributing to normal intestinal functionality. The species is not known to be involved in flavonoid biotransformation. Here, we report that the genomes of the species have homologs for various enzymes such as aryl-sulfate sulfotransferase, beta-glucuronidase, xylan 1,4-beta-xylosidase, alpha-L-rhamnosidase, quercetin-2,3-dioxygenase, 2-enoate reductase, caffeoyl-CoA_O-methyltransferase, arylsulfatase, alpha-glucosidase, beta-glucosidase, and sucrose phosphorylase, which suggest its potential ability to modify flavonoids in the gut. Similarly, *Klebsiella pneumoniae*, a Gram-negative bacterium widely recognized as an opportunistic pathogen causing a wide range of infections, has not yet been reported to have flavonoid transformation abilities. We found that the genomes of this species have homologs for alpha-L-rhamnosidase, quercetin-2,3-dioxygenase, beta-glucuronidase, beta-glucosidase, xylan 1,4-beta-xylosidase, alpha-glucosidase, arylsulfatase, protocatechuate 3,4-dioxygenase, and glutathionyl-hydroquinone reductase. Other species of the genus *Klebsiella*, including *Klebsiella aerogenes*, *Klebsiella variicola*, *Klebsiella quasipneumoniae*, *Klebsiella_A michiganensis*, *Klebsiella_A indica*, also have homologs for enzymes that can act upon flavonoids. *Olsenella_E sp003609875*, an anaerobic actinobacterium not reported to be involved in flavonoid metabolism, has homologs for Daidzein reductase and genistein reductase. *Faecalibacterium prausnitzii* is one of the most abundant commensals of the human colon. The species is not reported to be involved in flavonoid modification. Here we found that *Faecalibacterium prausnitzii_C* and *Faecalibacterium prausnitzii_D* have homologs for Flr (ANU40626.1). These two species also have homologs for alpha-glucosidase, beta-glucosidase, beta-glucuronidase, alpha-L-rhamnosidase, and xylan 1,4-beta-xylosidase.

The *Bacteroides* genus is the common inhabitant of the human gut. The genus includes Gram-negative anaerobes. *Bacteroides faecis* is a colonic commensal closely related to *Bacteroides thetaiotaomicron* [1]. The species is not yet reported to be involved in flavonoid modification. Here we found that the genomes of this species have homologs for alpha-L-rhamnosidase (WP_01110756), caffeoyl-CoA_O-methyltransferase, beta-glucosidase (AAO76887.1), quercetin-2,3-dioxygenase, and xylan 1,4-beta-xylosidase. Showing its flavonoid-modifying capabilities. *Bacteroides xylanisolvens* is known for its ability to degrade plant fibers such as xylan, but its flavonoid-modifying capacity has not been previously reported. However, we discovered that the species has homologs for several potential flavonoid-modifying enzymes, including alpha-L-rhamnosidase (WP_011107561), quercetin-2,3-dioxygenase, caffeoyl-CoA O-methyltransferase, beta-glucuronidase, xylan 1,4-beta-xylosidase, and beta-glucosidase (AAO76887.1). Several other *Bacteroides* species, including *Bacteroides ovatus, Bacteroides thetaiotaomicron, Bacteroides stercoris, Bacteroides sp902362375, Bacteroides sp900765785, Bacteroides intestinalis, Bacteroides cellulosilyticus, Bacteroides faecis, Bacteroides fragilis, Bacteroides eggerthii, and Bacteroides finegoldii*, also possess homologs for quercetin-2,3-dioxygenase, alpha-L-rhamnosidase, and xylan 1,4-beta-xylosidase. Interestingly, we also found that bacteria with experimentally known flavonoid metabolizing enzymes carry homologs for other potential flavonoid modifying genes. For example, *Bacteroides uniformis* species is involved in isoflavone degradation, and we identified homologs for several potential flavonoid-modifying enzymes in its genome, including alpha-L-rhamnosidase, quercetin-2,3-dioxygenase, caffeoyl-CoA O-methyltransferase, aryl-sulfate sulfotransferase, arylsulfatase, beta-glucuronidase, xylan 1,4-beta-xylosidase, alpha-glucosidase, beta-glucosidase, and ABC transporters. *Bifidobacterium adolescentis* is a gram-positive commensal of the human gut that produces sucrose phosphorylase, which can act upon flavonoids. We identified homologs of beta-glucosidase (KEF29323.1, A0A072MSE8, A0A072N4Q2), ary-sulfate sulfotransferase, beta-glucuronidase, xylan 1,4-beta-xylosidase, alpha-L-rhamnosidase, and beta-D-glucosideglucohydrolase (A0A072MRT2) in its genome. Other *Bifidobacterium* species, such as *Bifidobacterium pseudocatenulatum, Bifidobacterium catenulatum, Bifidobacterium infantis, Bifidobacterium sp002742445, Bifidobacterium dentium, Bifidobacterium scardovii, Bifidobacterium angulatum, Bifidobacterium breve, and Bifidobacterium bifidum*, also possess homologs for beta-glucosidases, alpha-L-rhamnosidase, and sucrose phosphorylase. *E. coli*, a facultative anaerobe in the human gut, is known to produce beta-glycosidase that can hydrolyze various substances [2], beta-glucuronidase that removes glucuronic acid from glucuronides [3], and pyrin-like proteins possessing quercetinase activity. For example, *E. coli* HGU-3 produces beta-glucuronidase that converts baicalin to baicalein [4]. We identified homologs for other flavonoid-modifying enzymes, such as glutathionyl-hydroquinone reductase, sucrose phosphorylase, aryl-sulfate sulfotransferase, xylan 1,4-beta-xylosidase, alpha-glucosidase, and arylsulfatase in *E. coli* genomes. *Flavonifractor plautii*, a major flavonoid-degrading bacterium encodes Phy, Chi, Flr, and Fcr. In this study, we reported that genomes of this species also have homologs for arylsulfate sulfotransferase, which can act upon quercetin, isoquercetin, and taxifolin. Moreover, *Eubacterium ramulus* is another species well-known for degrading flavonoids. We found that the genome of *Eubacterium_I ramulus* contains homologs for sucrose phosphorylase, beta-glucosidase, and alpha-L-rhamnosidase. Another species, *Eubacterium_I ramulus_A*, has homologs for dgpA, dgpC, and beta-glucosidase. These findings suggest that various bacteria in the human gut possess enzymes that can potentially metabolize flavonoids. Combining experimental approaches will provide insight into the role of unexplored bacterial species in the metabolism of various flavonoids.

**Supplementary Table 12: Gut bacterial species having homologs for potential flavonoid modifying proteins (At minimum 90 percent identity)**

| **UHGG Species Representative** | **Flavonoid modifying capacity from Literature search** | **EC Number/IDs of genes with homologs in species** | **Enzyme Name** |
| --- | --- | --- | --- |
| *Phocaeicola dorei* | NA | EC:1.13.11.24 | quercetin 2,3-dioxygenase |
|  |  | EC:1.3.1.31 | 2-enoate reductase |
|  |  | EC:2.1.1.104 | caffeoyl-CoA O-methyltransferase |
|  |  | EC:2.4.1.7 | sucrose phosphorylase |
|  |  | EC:2.8.2.22 | aryl-sulfate sulfotransferase |
|  |  | EC:3.1.6.1 | arylsulfatase (type I) |
|  |  | EC:3.2.1.20 | alpha-glucosidase |
|  |  | EC:3.2.1.21 | beta-glucosidase |
|  |  | EC:3.2.1.31 | beta-glucuronidase |
|  |  | EC:3.2.1.37 | xylan 1,4-beta-xylosidase |
|  |  | EC:3.2.1.40 | alpha-L-rhamnosidase |
| *Bifidobacterium adolescentis* | sucrose phosphorylase activity (De Winter et al. 2013), β-glucosidase activity, (Miyake, Yamamoto, and Osawa 1997; Raimondi et al. 2009) | KEF29323.1 | Beta-glucosidase BpBluA |
|  |  | EC:2.4.1.7 | sucrose phosphorylase |
|  |  | EC:2.8.2.22 | aryl-sulfate sulfotransferase |
|  |  | EC:3.2.1.20 | alpha-glucosidase |
|  |  | EC:3.2.1.21 | beta-glucosidase |
|  |  | EC:3.2.1.31 | beta-glucuronidase |
|  |  | EC:3.2.1.37 | xylan 1,4-beta-xylosidase |
|  |  | EC:3.2.1.40 | alpha-L-rhamnosidase |
|  |  | A0A072MRT2 | Beta-D-glucosideglucohydrolase |
|  |  | A0A072MSE8 | Beta-glucosidase |
|  |  | A0A072N4Q2 | Beta-glucosidase |
| *Klebsiella pneumoniae* | NA | EC:1.13.11.24 | quercetin 2,3-dioxygenase |
|  |  | EC:1.13.11.3 | protocatechuate 3,4-dioxygenase |
|  |  | EC:1.8.5.7 | glutathionyl-hydroquinone reductase |
|  |  | EC:3.1.6.1 | arylsulfatase (type I) |
|  |  | EC:3.2.1.20 | alpha-glucosidase |
|  |  | EC:3.2.1.21 | beta-glucosidase |
|  |  | EC:3.2.1.31 | beta-glucuronidase |
|  |  | EC:3.2.1.37 | xylan 1,4-beta-xylosidase |
|  |  | EC:3.2.1.40 | alpha-L-rhamnosidase |
| *Bacteroides uniformis* | β-glucosidase activity (Bokkenheuser, Shackleton, and Winter 1987; Miyake et al. 1997) | WP_011107561 | Alpha-L-Rhamnosidase BtR |
|  |  | EC:1.13.11.24 | quercetin 2,3-dioxygenase |
|  |  | EC:2.1.1.104 | caffeoyl-CoA O-methyltransferase |
|  |  | EC:2.8.2.22 | aryl-sulfate sulfotransferase |
|  |  | EC:3.1.6.1 | arylsulfatase (type I) |
|  |  | EC:3.2.1.20 | alpha-glucosidase |
|  |  | EC:3.2.1.21 | beta-glucosidase |
|  |  | EC:3.2.1.31 | beta-glucuronidase |
|  |  | EC:3.2.1.37 | xylan 1,4-beta-xylosidase |
|  |  | EC:3.2.1.40 | alpha-L-rhamnosidase |
|  |  | EC:7.6.2.2 | ABC-type xenobiotic transporter |
| *Bacteroides ovatus* | β-glucosidase activity (Bokkenheuser et al. 1987) | AAO76887.1 | Beta-glucosidase BtGlu (Periplasmic beta-glucosidase) |
|  |  | WP_011107561 | Alpha-L-Rhamnosidase BtR |
|  |  | EC:1.13.11.24 | quercetin 2,3-dioxygenase |
|  |  | EC:2.1.1.104 | caffeoyl-CoA O-methyltransferase |
|  |  | EC:2.8.2.22 | aryl-sulfate sulfotransferase |
|  |  | EC:3.2.1.20 | alpha-glucosidase |
|  |  | EC:3.2.1.21 | beta-glucosidase |
|  |  | EC:3.2.1.31 | beta-glucuronidase |
|  |  | EC:3.2.1.37 | xylan 1,4-beta-xylosidase |
|  |  | EC:3.2.1.40 | alpha-L-rhamnosidase |
| *Bacteroides faecis* | NA | AAO76887.1 | Beta-glucosidase BtGlu (Periplasmic beta-glucosidase) |
|  |  | WP_011107561 | Alpha-L-Rhamnosidase BtR |
|  |  | EC:1.13.11.24 | quercetin 2,3-dioxygenase |
|  |  | EC:2.1.1.104 | caffeoyl-CoA O-methyltransferase |
|  |  | EC:3.2.1.37 | xylan 1,4-beta-xylosidase |
|  |  | EC:3.2.1.40 | alpha-L-rhamnosidase |
| *Olsenella_E sp003609875* | NA | WP_013979957.1 | Daidzein reductase |
|  |  | A0A0U1WKA6 | Daidzein and genistein reductase |
| *Escherichia coli_D* | β-glucosidase activity (Hur et al. 2000), Quercetinase Activity (Adams and Jia 2005) | P46852 | Pyrin like protein (Quercetinase) |
|  |  | EC:1.8.5.7 | glutathionyl-hydroquinone reductase |
|  |  | EC:2.4.1.7 | sucrose phosphorylase |
|  |  | EC:2.8.2.22 | aryl-sulfate sulfotransferase |
|  |  | EC:3.1.6.1 | arylsulfatase (type I) |
|  |  | EC:3.2.1.20 | alpha-glucosidase |
|  |  | EC:3.2.1.21 | beta-glucosidase |
|  |  | EC:3.2.1.31 | beta-glucuronidase |
|  |  | EC:3.2.1.37 | xylan 1,4-beta-xylosidase |
| *Bacteroides xylanisolvens* | NA | AAO76887.1 | Beta-glucosidase BtGlu (Periplasmic beta-glucosidase) |
|  |  | WP_011107561 | Alpha-L-Rhamnosidase BtR |
|  |  | EC:1.13.11.24 | quercetin 2,3-dioxygenase |
|  |  | EC:2.1.1.104 | caffeoyl-CoA O-methyltransferase |
|  |  | EC:3.2.1.31 | beta-glucuronidase |
|  |  | EC:3.2.1.37 | xylan 1,4-beta-xylosidase |
|  |  | EC:3.2.1.40 | alpha-L-rhamnosidase |
| *Faecalibacterium prausnitzii_C* | NA | ANU40626.1 | Flavone/flavonol reductase (Flr) |
|  |  | EC:1.1.1.219 | dihydroflavonol 4-reductase |
|  |  | EC:3.2.1.20 | alpha-glucosidase |
|  |  | EC:3.2.1.21 | beta-glucosidase |
|  |  | EC:3.2.1.31 | beta-glucuronidase |
|  |  | EC:3.2.1.37 | xylan 1,4-beta-xylosidase |
|  |  | EC:3.2.1.40 | alpha-L-rhamnosidase |
| *Flavonifractor plautii* | CHI, Flr, Fcr, and PHY (Goris et al., 2021; Kutschera et al., 2011; Yang et al., 2021) | ANU40626.1 | Flavone/flavonol reductase (Flr) |
|  |  | EHM54434.1 | Chalcone isomerase (CHI) |
|  |  | WP_154024723.1 | Flavanone/flavanonol-cleaving reductase (Fcr) |
|  |  | EC:2.8.2.22 | aryl-sulfate sulfotransferase |
|  |  | EC:3.7.1.4 | PHY |
| *Eubacterium_I ramulus* | Fcr, PHY, CHI | AGS82961.1 | Flavanone/flavanonol-cleaving reductase (Fcr) |
|  |  | EC:2.4.1.7 | sucrose phosphorylase |
|  |  | EC:3.2.1.21 | beta-glucosidase |
|  |  | AIS36173.1 | CHI |
|  |  | EC:3.2.1.40 | alpha-L-rhamnosidase |
|  |  | EC:3.7.1.4 | PHY |

**Supplementary text 2**

**Distribution of biochemically characterized FMEs in the UHGG species**

We analyzed the distribution of UHGP homologs for biochemically characterized FMEs in the genomes of the UHGG catalog by locating these homologs in the UHGG metadata table. A species was considered to have flavonoid-metabolizing potential if any one genome within that species contained homologs for a particular FME.

Homologs for sequence (BBG22493.1) that catalyses C-deglycosylation of isoflavones were present in the genomes of 755 species. In contrast, other sequences associated with C-deglycosylation, including BBG22495.1 and BBG22494.1, were found to have a lower number of hits and were present in genomes of 75 and 73 species, respectively. Homologs for EIM58369.1 that involved in C-deglycosylation of flavones were present in genomes of 512 species while other sequences related to this step exhibited lower prevalence, with EIM58373.1 found in genomes of 36 species, EIM58371.1 in 84 species, EIM58372.1 in 77 species, and EIM58370.1 in 15 species. Homologs of KEF29323.1 that catalyses 7-o-deglycosylation of isoflavones were also of lower prevalence and found in genomes of 30 species. Similarly, homologs associated with o-deglycosylation FMEs were of lower prevalence, with sequences A0A072MRT2 found in 32 species, A0A072MSE8 in 28 species, and A0A072N4Q2 in 21 species. Moreover, homologs of AKC35075.1 and AKC35076.1, involved in 7-O-deglycosylation of isoflavones and flavones, were identified in genomes of 415 and 30 species, respectively. homologs for WP_011107561 (Alpha-L- rhamnosidase) were found in genomes of 84 species.

FMEs associated with flavonol/flavone degrading pathways were prevalent in gut bacterial genomes. For example, homologs of flr (ANU40626.1, ADK16070.1) were found in genomes of over 133 species. Homologs of two fcr sequences, AGS82961.1 and WP_154024723.1, were identified in 254 and 150 species, respectively. Hits for CHI were observed in 40 species for EHM54434.1 and in 16 species for AIS36173.1. Homologs for phy were found in 20 species for AAQ12341.1 and OXE48401.1, six species for EUA80835.1, and 6 species for B1MK49. Genomes having homologs for all four key genes of flavonol/flavone degradation pathway are rare and highlighted in **Supplementary Table 13**.

FMEs associated with the eqol formation pathway were abundant in gut bacterial species. Hits for Daidzein reductase were observed in 26 species for BAJ22678.1, 21 species for AFV15453.1 and BAL46930.1, 24 species for A0A0U1WKA6, and three species for WP_013979957.1 detected. Homologs for Dihydrodaidzein reductase (BAL46929.1) and WP_013979959.1 were widespread, being found in 1,202 and 876 species, respectively. Additionally, dihydrodaidzein reductase (BAJ72745.1) was identified in 616 species, while BAM25050.1 had a more limited distribution, being found in only nine species. Hits for Tetrahydrodaidzein reductase BAL46928.1 were detected in 175 species, with BAJ72744.1 and AFV15450.1 present in 183 and 133 species, respectively, and WP_013979960.1 was found in 68 species. Homologs of FMEs involved in methyl transfer, such as Corrinoid protein and Methyltransferase-II, were widely distributed, being present in many species (more than 1000). Hits for ANI69959.1 were identified in 216 species, and for ANI69960.1 were found in 27 species. Hits for 4-coumarate_3-hydroxylase (Q2EYY8) were detected in 78 species, Glycosyl transferase (Q65JC2) in 18 species, arylsulfate sulfotransferase (B8FRJ0) in three species, and Dextransucrase (Q9ZAR4) in three species.

**Supplementary Table 13. Gut bacterial species having homologs for key genes of flavonol/flavone degradation pathway**

| **Species representative** | **MGnify ID** | **Homologs for flavone/flavonol degradation pathway** |
| --- | --- | --- |
| *Eubacterium_I sp900066595* | MGYG000000058 | ANU40626.1,  EHM54434.1, fpla:A4U99_15225,  WP_154024723.1,  ag:AAQ12341,  AGS82961.1,  AIS36173.1,  ADK16070.1 |
| *Eubacterium_I ramulus* | MGYG000001456.1 | AGS82961.1  ANU40626.1  AIS36173.1  AAQ12341.1  ADK16070.1 |
| *Flavonifractor plautii* | MGYG000000099 | ANU40626.1,  EHM54434.1, fpla:A4U99_15225,  WP_154024723.1,  ADK16070.1,  AGS82961.1 |
| *Eubacterium_I sp900546495* | MGYG000000969 | ANU40626.1,  WP_154024723.1,  ag:AAQ12341,  AGS82961.1,  AIS36173.1,  ADK16070.1 |

## **Supplementary text 3.**

114 flavonoid-related keywords obtained from Ivey et al were searched as query in BRENDA and KEGG databases, these includes; cyanidin, flavylium, ideain, kuromanin, keracyanin, delphinidin, malvidin, malvin, pelargonidin, peonidin, petunidin, pigment, pinotin, vitisin, butein, chalcone, phloretin, phloridzin, myricetin, quercetin, astilbin, catechin, flavanone, flavane, taxifolin, distylin, cinnamtannin, tetramer d, flavin, bonannione, mimulone, naringenin, flavanone, bavachin, didymin, eriodictyol, hesperetin, hesperidin, xanthohumol, prunin, narirutin, eriocitrin, chrysin, pinocembrin, poncirin, sakuranetin, flavone, luteolin, citrifolioside, apigenin, vicenin, vitexin, apiin, baicalein, chrysoeriol, eupatorin, diosmin, cirsimaritin, cirsilineol, scutellarein, skrofullein, diosmetin, gardenin, geraldone, hispidulin, rhoifolin, jaceosidin, orientin, scolymoside, nepetin, nobiletin, pebrellin, rhoifolin, tangeretin, scutellarein, sinensetin, kaempferol, galangin, rhamnetin, jaceidin, kaempferide, trifolin, astragalin, nicotiflorine, morin, patuletin, avicularin, hyperoside, hyperin, quercitrin, quercetrin, rutin, reynoutrin, spiraeoside, spinacetin, genisteol, neochanin, pratol, daidzin, genistin, glycitin, genistein, pratensol, biochanin, daidzein, daidzeol, formononetin, prunetol, sophoricol, glycitein, flavonoid, flavonol, flavanol, anthocyan.

**Supplementary text 4**

To identify homologs of bacterial protein sequences (30,598 sequences obtained from KEGG and BRENDA) in the gut bacterial protein catalog (UHGP) [5], we conducted a blastp analysis using Diamond software. The protein sequences obtained from UHGP were aligned to the protein sequences database created using 30,601 bacterial sequences.  We applied three different criteria to select the best hits. The first criterion was set at 80% coverage, 90% percent identity (PID), and 60-bit score, which resulted in the identification of 3,048 proteins with homologs in genomes of 1,242 species representatives. However, some proteins that share a lower percent identity but possess the potential to carry out flavonoid modifications could be missing [6]. For example, the chalcone isomerase from *F. plautii* shares only 50% sequence identity with the CHI from *E. ramulus*, and the FLR protein of *Clostridium ljungdahlii* shares 29.69% amino acid identity with the FLR protein of *F. plautii*. Despite these lower sequence identities, these enzymes demonstrate flavonoid-metabolizing functions.[7,8].

We subsequently used the parameters of the eggnog mapper, which were set at 20% coverage, 40% PID, and 60-bit score, resulting in the identification of 7,341 protein sequences with homologs in genomes of 4,514 gut bacterial species representatives. Finally, we used the 75% coverage and 30% PID criteria, as used by Goris et al. (2021), and identified 6,865 prokaryotic protein sequences with homologs in the genomes of 4,606 representative gut bacterial species [9]. After carefully evaluating the results, we selected this criterion (30% PID, 75% coverage, 60-bit score) for our final analysis.

**
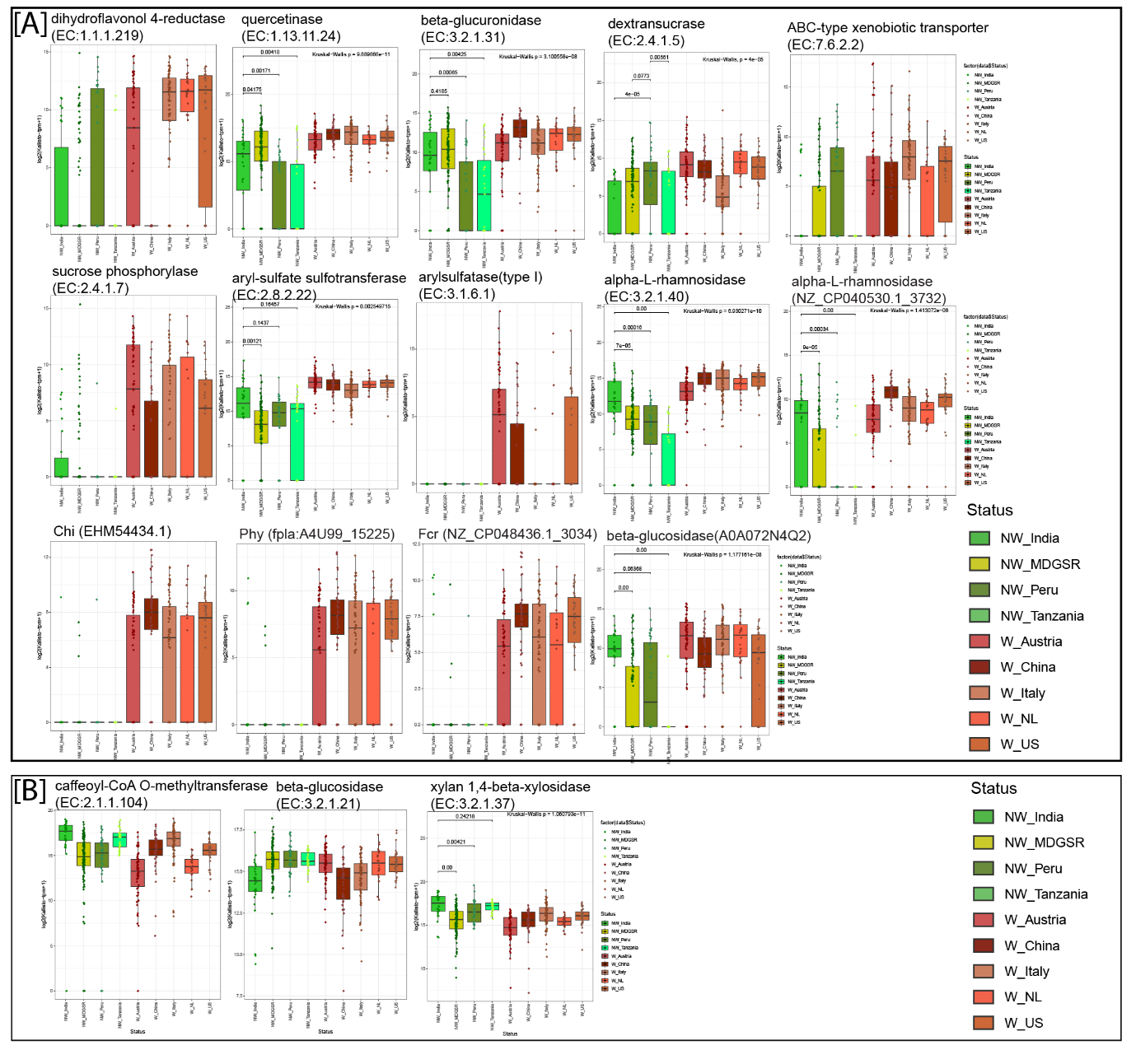
**

**Supplementary Figure 1**. **Boxplots showing differentially abundant potential flavonoid modifying ECs and genes based on log(tpm+1) (Boruta-selected 207 genes mapped to their ECs).** Boxplots showing differentially abundant potential flavonoid modifying ECs and genes in Western **(A)** and non-Western **(B)** populations. Kruskal Wallis test was used for comparing multiple groups, and the Wilcoxon rank-sum test was used to calculate p values.

**
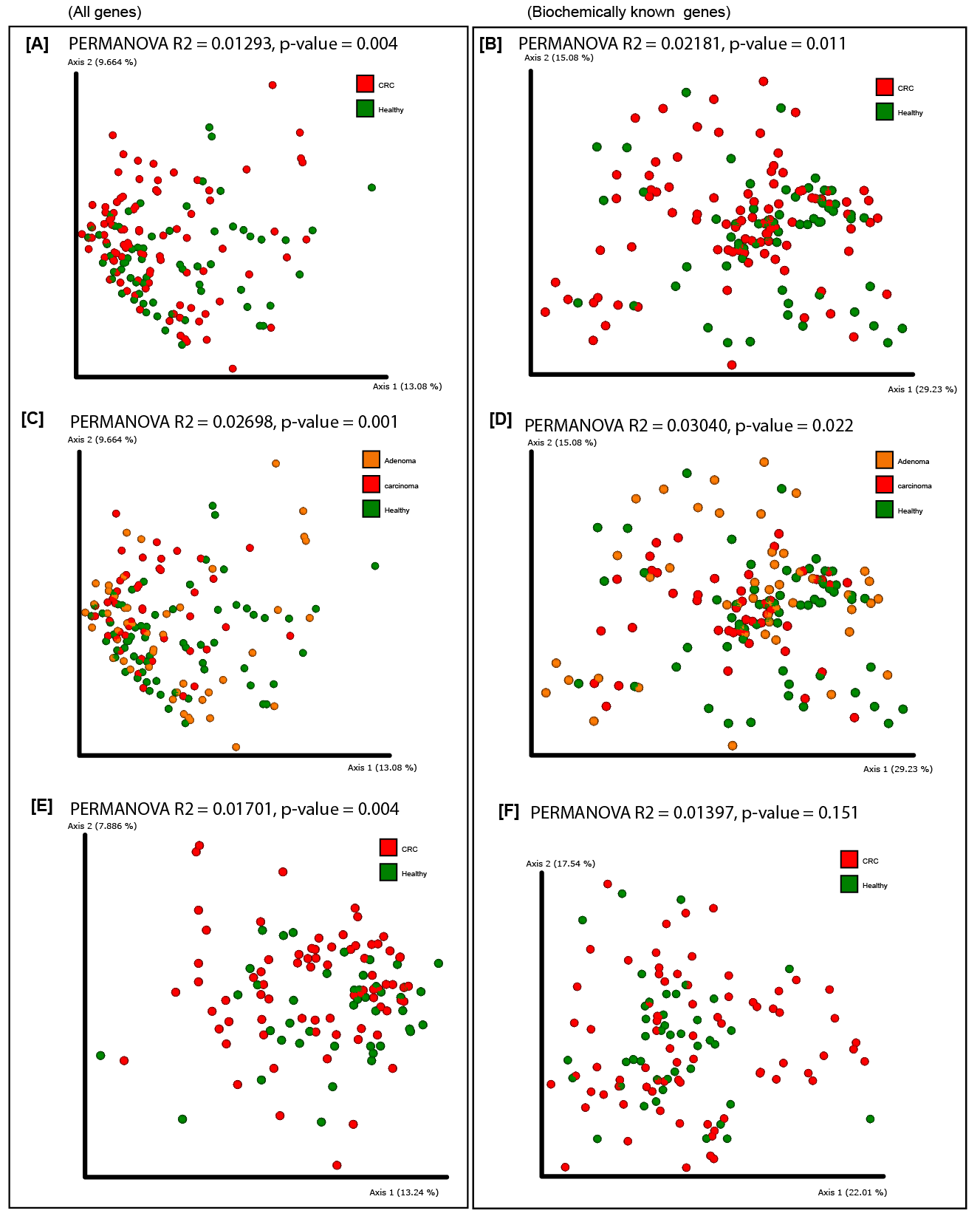
**

**Supplementary Figure 2**. **PCoA showing the distribution of potential flavonoid modifying genes based on inter-sample Bray-Curtis distance calculated using (log(tpm+1))**. PCoA showing the distribution of all genes **(A)** and for biochemically characterized genes **(B)** in healthy and CRC samples of Austrian population. PCoA showing the distribution of genes in healthy, adenoma, and carcinoma samples of Austrian population for all genes **(C)** and for biochemically characterized genes **(D)**. PCoA showing the distribution of Genes in healthy and CRC samples of Chinese population for all genes **(E)** and biochemically characterized genes **(F)**.


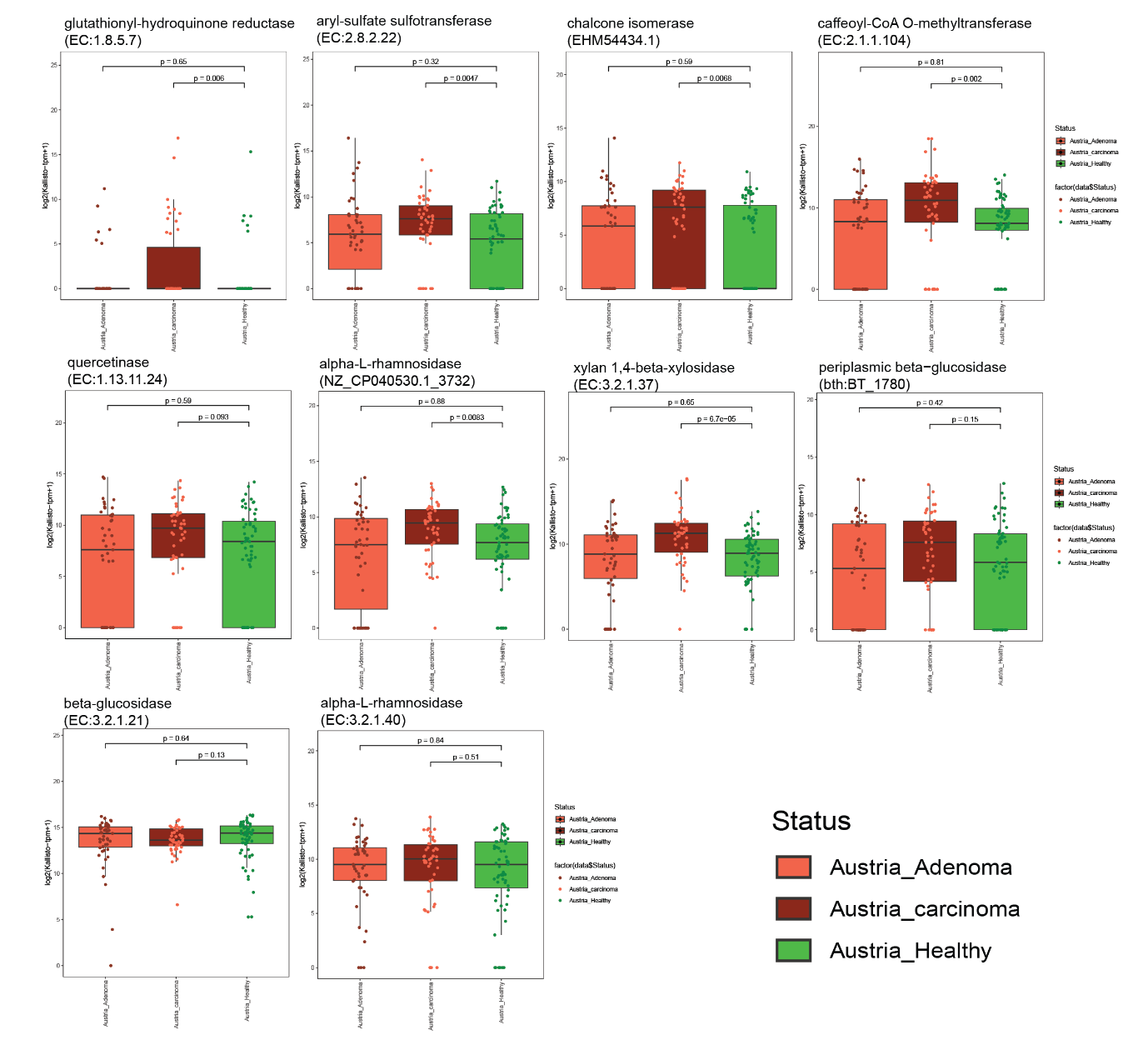


**Supplementary Figure 3**. **Boxplots showing differentially abundant potential flavonoid modifying ECs and genes based on log(tpm+1).** Boruta-selected 28 genes in Indian population were mapped to their ECs and their abundance was plotted in Adenoma vs Healthy and Carcinoma vs Healthy groups of Austrian population.

**
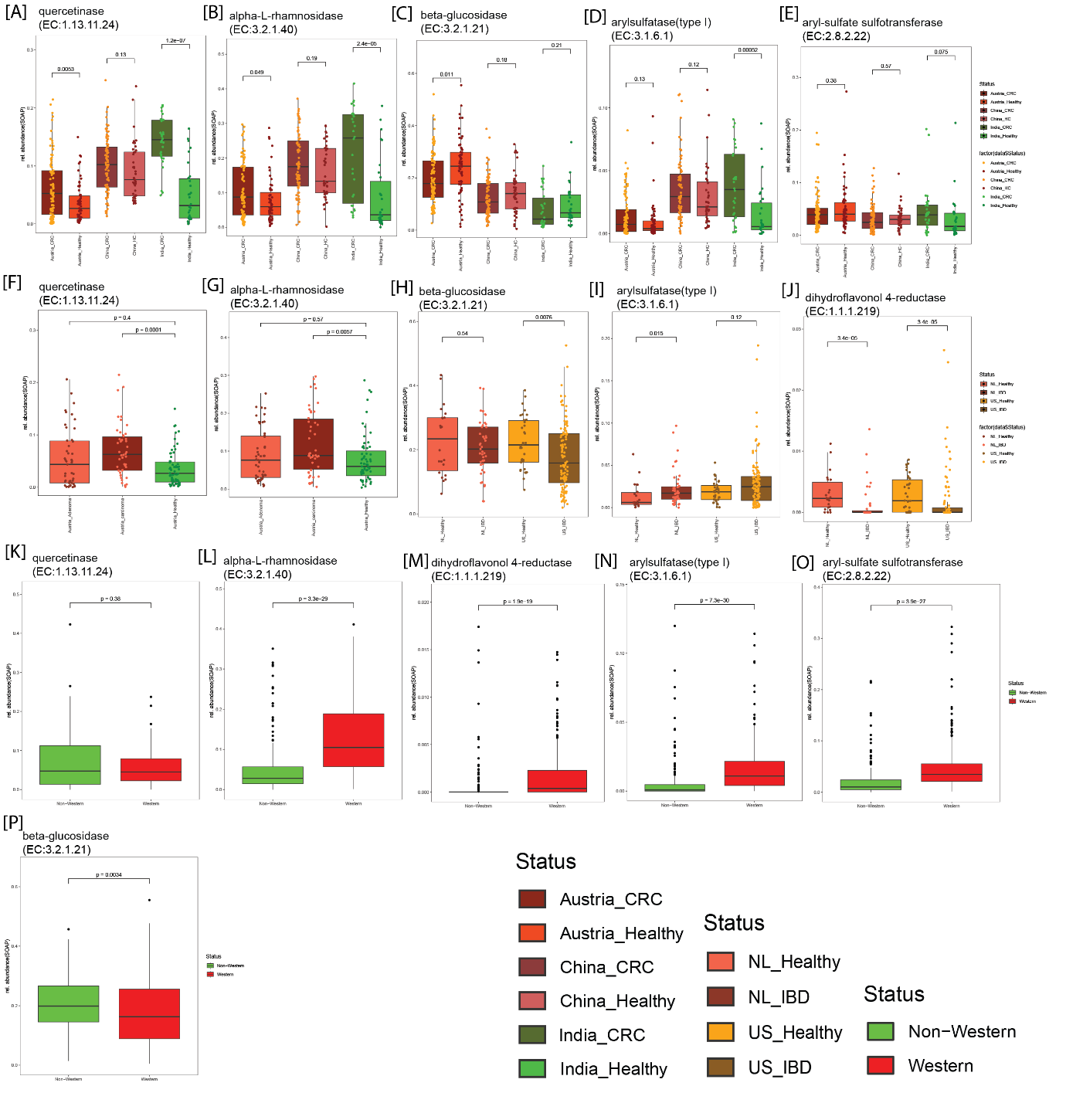
**

**Supplementary Figure 4.** **Boxplots based on relative abundance (calculated using SOAP)**. Boxplots showing differentially abundant ECs in Healthy vs. CRC samples of Austrian, Chinese, and Indian populations **(A-E)**. Boxplots showing differentially abundant ECs in Healthy vs. Adenoma, Healthy vs. Carcinoma in Austrian populations, and Healthy vs. IBD samples in the US and Netherlands populations **(F-J)**. Boxplots showing differentially abundant ECs in Healthy Western vs. Healthy Non-Western populations **(K-P)**. The whiskers, bounds of the box, and the line in the middle of the box represent the min-to-max values, 25th–75th percentiles, and median, respectively. Wilcoxon rank-sum test was used to calculate p-values.

**
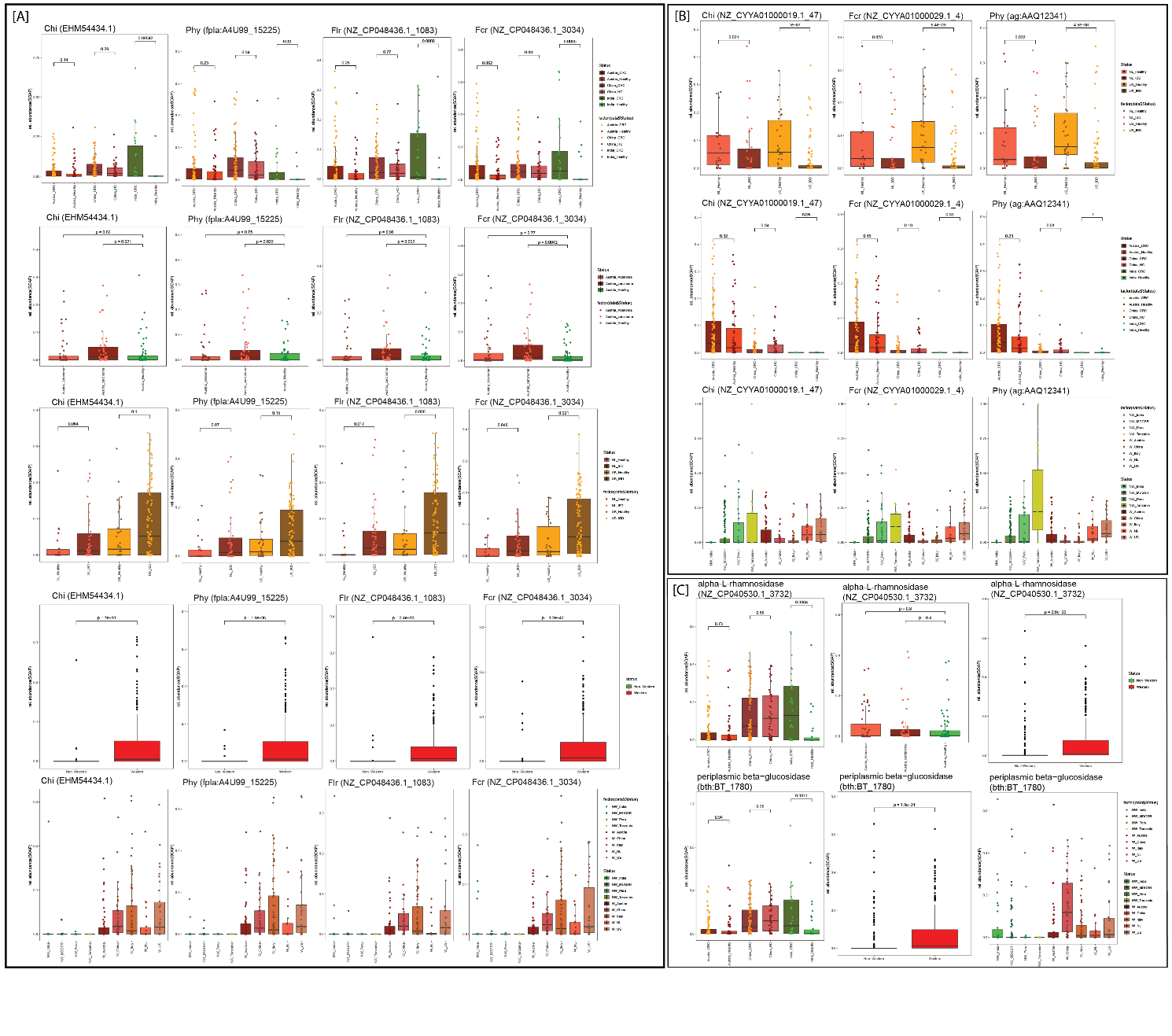
**

**Supplementary Figure 5.** **Boxplots based on relative abundance (calculated using SOAP**. Boxplots showing differentially abundant flavonoid metabolizing genes encoding Chi (EHM54434.1), Phy (fpla:A4U99_15225), Flr (NZ_CP048436.1_1083), and Fcr (NZ_CP048436.1_3034) in Healthy vs. CRC samples of Austrian, Chinese, and Indian populations, Healthy vs. Adenoma and Healthy vs. Carcinoma samples of Austrian population, Healthy vs. IBD samples of US and Netherlands (NL) populations, and Healthy Western vs. Healthy Non-Western populations **(A)**. Boxplots showing differentially abundant flavonoid metabolizing genes encoding Chi (NZ_CYYA01000019.1_47), Fcr (NZ_CYYA01000029.1_4), and Phy (ag:AAQ12341) in Healthy vs. IBD samples of US and Netherlands populations, and boxplots showing lower abundance of these genes in both Healthy and CRC samples from Indian compared to other populations **(B).** Boxplots showing differentially abundant periplasmic beta-glucosidase (bth:BT_1780) and alpha-L-rhamnosidase (NZ_CP040530.1_3732) in Healthy vs. CRC samples of Austrian, Chinese, and Indian populations; Healthy vs. Adenoma and Healthy vs. Carcinoma samples from Austrian populations; and Healthy Western vs. Healthy Non-Western populations **(C)**. The whiskers, bounds of the box, and the line in the middle of the box represent the minimum to maximum values, 25th to 75th percentiles, and median, respectively. The Wilcoxon rank-sum test was used to calculate p-values.

**References:**

1. Kim, M.S.; Whon, T.W.; Roh, S.W.; Shin, N.R.; Bae, J.W. Draft Genome Sequence of Bacteroides Faecis MAJ27 T, a Strain Isolated from Human Feces. *J. Bacteriol.* **2011**, *193*, 6801–6802, doi:10.1128/JB.06210-11.

2. Rodríguez-Daza, M.C.; Pulido-Mateos, E.C.; Lupien-Meilleur, J.; Guyonnet, D.; Desjardins, Y.; Roy, D. Polyphenol-Mediated Gut Microbiota Modulation: Toward Prebiotics and Further. *Front. Nutr.* **2021**, *8*, 347, doi:10.3389/FNUT.2021.689456/BIBTEX.

3. Candeliere, F.; Raimondi, S.; Ranieri, R.; Musmeci, E.; Zambon, A.; Amaretti, A.; Rossi, M. β-Glucuronidase Pattern Predicted From Gut Metagenomes Indicates Potentially Diversified Pharmacomicrobiomics. *Front. Microbiol.* **2022**, *13*, 267, doi:10.3389/FMICB.2022.826994/BIBTEX.

4. Kim, D.H.; Jang, I.S.; Lee, H.K.; Jung, E.A.; Lee, K.Y. Metabolism of Glycyrrhizin and Baicalin by Human Intestinal Bacteria. *Arch. Pharm. Res.* **1996**, *19*, 292–296, doi:10.1007/BF02976243/METRICS.

5. Almeida, A.; Nayfach, S.; Boland, M.; Strozzi, F.; Beracochea, M.; Shi, Z.J.; Pollard, K.S.; Sakharova, E.; Parks, D.H.; Hugenholtz, P.; et al. A Unified Catalog of 204,938 Reference Genomes from the Human Gut Microbiome. *Nat. Biotechnol.* **2021**, *39*, 105–114, doi:10.1038/s41587-020-0603-3.

6. Pearson, W.R. An Introduction to Sequence Similarity (“Homology”) Searching. **2013**, doi:10.1002/0471250953.bi0301s42.

7. Yang, G.; Hong, S.; Yang, P.; Sun, Y.; Wang, Y.; Zhang, P.; Jiang, W.; Gu, Y. Discovery of an Ene-Reductase for Initiating Flavone and Flavonol Catabolism in Gut Bacteria. *Nat. Commun.* **2021**, *12*, 1–15, doi:10.1038/s41467-021-20974-2.

8. Braune, A.; Engst, W.; Elsinghorst, P.W.; Furtmann, N.; Bajorath, J.; Gütschow, M.; Blaut, M. Chalcone Isomerase from Eubacterium Ramulus Catalyzes the Ring Contraction of Flavanonols. *J. Bacteriol.* **2016**, *198*, 2965–2974, doi:10.1128/JB.00490-16.

9. Goris, T.; Cuadrat, R.R.C.; Braune, A. Flavonoid-Modifying Capabilities of the Human Gut Microbiome—an in Silico Study. *Nutrients* **2021**, *13*, doi:10.3390/nu13082688.
